# Characteristics of the Fatty Acid Composition in Elderly Patients with Occupational Pathology from Organophosphate Exposure

**DOI:** 10.3390/diagnostics15243246

**Published:** 2025-12-18

**Authors:** Nikolay V. Goncharov, Elena I. Savelieva, Tatiana A. Koneva, Lyudmila K. Gustyleva, Irina A. Vasilieva, Mikhail V. Belyakov, Natalia G. Voitenko, Daria A. Belinskaia, Ekaterina A. Korf, Richard O. Jenkins

**Affiliations:** 1 Research Institute of Hygiene, Occupational Pathology and Human Ecology of the Federal Medical Biological Agency, 188663 Kuzmolovsky, Russia; 2 Sechenov Institute of Evolutionary Physiology and Biochemistry, Russian Academy of Sciences, 194223 Saint Petersburg, Russia; 3 Department of Biological Chemistry, St. Petersburg State Pediatric Medical University, 194100 Saint Petersburg, Russia; 4 School of Allied Health Sciences, De Montfort University, The Gateway, Leicester LE1 9BH, UK

**Keywords:** age-related diseases, organophosphates, occupational pathology, esterified and non-esterified fatty acids, omega-3 fatty acids, biomarker

## Abstract

**Background/Objectives:** The delayed effects of organophosphate poisoning may manifest years after exposure, often masked by age-related diseases. The aim of this retrospective cohort study was to identify the biochemical “trace” that could remain in patients decades after poisoning. We determined a wide range of biochemical parameters, along with the spectrum of esterified and non-esterified fatty acids (EFAs and NEFAs, respectively), in the blood plasma of a cohort of elderly patients diagnosed with occupational pathology (OP) due to (sub)chronic exposure to organophosphates in the 1980s. **Methods:** Elderly patients with and without a history of exposure to organophosphates were retrospectively divided into two groups: controls (*n* = 59, aged 73 ± 4, men 29% and women 71%) and those with OP (*n* = 84, aged 74 ± 4, men 29% and women 71%). The period of neurological examination and blood sampling for subsequent analysis was from mid-2022 to the end of 2023. Determination of the content of biomarkers of metabolic syndrome, NEFAs, and EFAs in blood plasma was performed by HPLC-MS/MS and GC-MS. **Results:** The medical histories of the examined elderly individuals with OP and the aged control group included common age-related diseases. However, patients with OP more often had hepatitis, gastrointestinal diseases, polyneuropathy, and an increased BMI. Analysis of metabolic biomarkers revealed, in the OP group, a decrease in the concentrations of 3-hydroxybutyrate (*p* < 0.05), 2-hydroxybutyrate (*p* < 0.0001), and acetyl-L-carnitine (*p* < 0.001) and the activity of butyrylcholinesterase (BChE) (*p* < 0.05), but an increase in the esterase activity of albumin (*p* < 0.05). Correlation analysis revealed significant relationships between albumin esterase activity and arachidonic acid concentrations in the OP group (0.64, *p* < 0.0001). A study of a wide range of fatty acids in patients with OP revealed reciprocal relationships between EFAs and NEFAs. A statistically significant decrease in concentration was shown for esters of margaric, stearic, eicosadienoic, eicosatrienoic, arachidonic, eicosapentaenoic, and docosahexaenoic fatty acids. A statistically significant increase in concentration was shown for non-esterified heptadecenoic, eicosapentaenoic, eicosatrienoic, docosahexaenoic, γ-linolenic, myristic, eicosenoic, arachidonic, eicosadienoic, oleic, linoleic, palmitic, linoelaidic, stearic, palmitoleic, pentadecanoic, and margaric acids. Decreases in the ratios of omega-3 to other unsaturated fatty acids were observed only for the esterified forms. **Conclusions:** The data obtained allow us to consider an increased level of NEFAs as one of the main cytotoxic factors for the vascular endothelium. Modification of albumin properties and decreased bioavailability of docosahexaenoic acid could be molecular links that cause specific manifestations of organophosphate-induced pathology at late stages after exposure.

## 1. Introduction

Organophosphates are among the most common xenobiotics, the neurotoxic effects of which have been thoroughly investigated [1,2]. Every year, millions of people worldwide become victims of accidental poisoning by pesticides, organophosphate-containing flame retardants, and environmental pollutants [3,4,5,6]. A significant proportion of these are workers in industrial and agricultural enterprises, whose poisoning often occurs as a result of non-compliance with safety regulations. Four conditions caused by the neurotoxic effects of organophosphates are usually identified: cholinergic crisis, intermediate syndrome, organophosphate-induced delayed polyneuropathy, and chronic organophosphate exposure disorder [7]. This list can be supplemented by Gulf War syndrome [8]. According to numerous studies, organophosphate exposure in humans can lead to aberrations in embryonic development; defects in neurocognitive function in early life; and a significant contribution to the development of neurodegenerative diseases in adults [9,10,11,12,13]. At least two of the above-mentioned conditions of organophosphate-induced pathology depend on the severity of acute poisoning and manifest in the short term.

The clinical symptoms and diagnostic criteria of cholinergic syndrome are described in many articles on toxicology [7]. They are associated primarily with the suppression of synaptic acetylcholinesterase activity, the subsequent accumulation of acetylcholine, and, as a result, the hyperactivation of nicotinic and muscarinic receptors. The early phase of convulsive activity (the cholinergic phase of the toxicogenic stage of poisoning, up to 5 min after the onset of the attack) initially involves mixed cholinergic and non-cholinergic modulation (5–40 min) and then transforms into the non-cholinergic phase of the toxicogenic stage of poisoning [14]. Intermediate syndrome occurs with a frequency of 7.7% to 84% within 1–4 days after acute poisoning with certain organophosphates, when the symptoms of cholinergic syndrome no longer appear. This condition results from damage to the neuromuscular junctions of the diaphragm, intercostal muscles, neck flexors, shoulder abductors, and hip flexors. At the molecular and ultrastructural levels, the development of intermediate syndrome and associated myopathy is associated with prolonged inhibition of acetylcholinesterase, desensitization and decreased expression of nicotinic receptors, oxidative stress, and muscle fiber necrosis [15]. The severity of the syndrome may be aggravated by endothelial damage as a result of measures taken in intensive care units [16]. Delayed polyneuropathy may appear 10–20 days after a single instance of acute poisoning with pronounced cholinergic syndrome, but tens of thousands of cases of delayed polyneuropathy in the USA, Morocco, Romania, Sri Lanka, China, and the former Yugoslavia are associated with repeated poisoning with triorthocresyl phosphate as part of surrogate alcohol or food products, without obvious signs of cholinergic syndrome [7]. The occurrence of delayed consequences of (sub)chronic intoxication with organophosphates is usually not associated with the suppression of acetylcholinesterase activity, and the consequences may include neurodegenerative diseases such as Parkinson’s disease, Alzheimer’s disease, and multiple sclerosis [17]. The development of Gulf War syndrome is associated with the combined effects of pyridostigmine bromide and permethrin, which were regularly taken by coalition combatants as a preventative measure against actual poisoning with highly toxic organophosphates and scabies/pediculosis, respectively [8]. Taking into account prophylactic vaccination and subsequent therapeutic intervention, several molecular mechanisms for the pathogenesis of Gulf War syndrome have been proposed, including chronic inflammation, oxidative stress, lipid and intestinal microbiota imbalances, and epigenetic modifications [18].

The remaining consequences of subchronic and chronic poisoning usually manifest years after exposure. In such cases, the complexity of diagnosis is aggravated by the aging of the body and comorbid age-related diseases, the differential diagnosis of which is an independent problem regardless of the toxicity factor [19]. Damage to the endothelia of blood vessels is one of the main pathogenetic factors of age-related diseases, including neurodegenerative diseases [20,21], and the pathogenesis of organophosphate-induced delayed peripheral polyneuropathy is associated with damage to endoneurial capillaries [22]. However, the question of cause-and-effect relationships and secondary mediators of the remaining pathology, arising as a result of the impact or manifestation of factors of a genetic, epigenetic, and sociodemographic nature, remains open. Such factors include lifestyle and diet, manifested in biochemical parameters of the blood, among which non-esterified fatty acids (NEFAs) and their acylated derivatives (esterified fatty acids, EFAs) play a significant but poorly studied role. Moreover, although hyperglycemia is the main risk factor for polyneuropathy in type 1 diabetes, it is known that metabolic syndrome and concomitant dyslipidemia underlie the occurrence and progression of polyneuropathy in type 2 diabetes and its prediabetic state [23,24].

There are studies indicating an important role for polyunsaturated fatty acids (PUFAs) in all-cause mortality, in the development of cardiovascular diseases, and in dementia [25,26]. Fatty acids are present in tissues and the bloodstream as part of complex lipids such as triglycerides and phospholipids, while NEFAs circulate in an unbound form. The hydrolysis of dietary lipids to NEFAs occurs before their absorption and subsequent involvement in lipid biosynthesis [27]. Thus, nutritional factors largely determine the fatty acid content in the blood. However, serum/plasma fatty acid profiles not only correlate with such indicators as body mass index (BMI) and muscle strength [28] but also show great indicative potential in the diagnosis of a number of diseases [29].

The aim of the research reported here was to identify the biochemical “trace” that could remain in patients decades after poisoning. For its implementation, we determined a wide range of biochemical parameters, along with the spectrum of fatty acids and their acylated derivatives, in the blood plasma of a unique cohort of elderly patients—former employees of a chemical enterprise (Novocheboksarsk, Russia) diagnosed with occupational pathology caused by subacute or (sub)chronic exposure to organophosphates in the 1980s. We proceeded from the assumption that the intoxication of the body with organophosphates followed by drug load causes systemic damage to vascular endothelial cells, the liver, and enterocytes and an imbalance in the intestinal microbiota, which serves as a trigger for metabolic diseases and is manifested in a persistent change in the lipid profile.

## 2. Materials and Methods

### 2.1. Study Design and Setting

Elderly patients (68 to 84 years old) with and without a history of exposure to organophosphates, who worked in the 1980–1990s at chemical industry enterprises, currently residing in the city of Novocheboksarsk and registered with Medical Unit 29 of the Federal Medical and Biological Agency, were retrospectively divided into two groups: a control group (*n* = 59) and a group with occupational pathology (*n* = 84). The period of patient group formation, their neurological examination, and blood sampling for subsequent analysis was approximately one and a half years—from mid-2022 to the end of 2023. The study was approved by the Ethics Committee of the Research Institute of Hygiene, Occupational Pathology, and Human Ecology of the Federal Medical Biological Agency (Approval No. 3, registration date 2 June 2022).

### 2.2. Eligibility Criteria

For both the control and OP groups, the inclusion criteria included data on the place and duration of employment, annual medical examination data, and registered cases of medical treatment for subacute or subchronic organophosphate poisoning during work in chemical industry enterprises. Exclusion criteria included data on progressive cancer and recent infectious diseases.

### 2.3. Outcomes and Measures

Structured interviews were conducted to obtain sociodemographic data. For elderly patients, medical history and neurological examination data were collected. After blood collection from representatives of all three groups, initial sample preparation and general biochemical analysis were performed, followed by the determination of a group of biomarkers for metabolic syndrome and fatigue, as well as esterified and non-esterified fatty acids in the blood plasma.

#### 2.3.1. Reagents and Standards

Acetonitrile, formic acid, ammonium formate, aqueous ammonia (25–27%), sulfuric acid, potassium dichromate, sodium hydroxide, gaseous nitrogen, and *o*-phosphoric acid—purchased from Lenreaktiv, St. Petersburg, Russia; meldonium dihydrate (pharmacopeial standard in the form of a powder substance)—purchased from SPC KEM, St. Petersburg, Russia; *n*-hexane, special purity grade—purchased from Kriokhrom, St. Petersburg, Russia; phosphate saline buffer tablets pH 7.2–7.6—purchased from Eco-Service LLC, St. Petersburg, Russia; methanol for gradient HPLC—purchased from Avantor™ Performance Materials (Phillipsburg, NJ, USA); methanol 99.9%—purchased from CARLO ERBA Reagents srl (Cornaredo, Italy); uric acid (≥99%), creatinine (≥99%), creatine (analytical grade), sodium 2-hydroxybutyrate (≥97%), 3-hydroxybutyrate (95%), acetylcarnitine hydrochloride, threonine (≥98%), inosine (≥99%), adenosine (≥99%), lactic acid (≥98%), tryptamine (≥98%), 3-methylhistidine, 2-hydroxymethylbutyrate (99%), 3-hydroxymethylbutyrate (≥95%), hypoxanthine (≥99%), uridine (≥99%), sodium methylate (>97%), tetrabutylammonium hydroxide 1 M solution in methanol, methyl iodide (≥99%), and deuterated palmitic acid D31—purchased from Sigma-Aldrich (St. Louis, MO, USA); deuterated methyl ester of palmitic acid D31—CDN Isotopes, Cat. No. D-1360; a mixture of methyl esters with certified content of 37 fatty acids (F.A.M.E.) C4–C24—purchased from Supelco, Merck KGaA (Darmstadt, Germany); methylene chloride (99.9%)—purchased from LiChrosolv, Merck KGaA (Darmstadt, Germany).

#### 2.3.2. Blood Collection and Plasma Preparation

Blood samples were collected, processed, and stored in accordance with international guidelines [30]. Blood was collected from subjects on an empty stomach from the cubital vein into BD Vacutainer vacuum tubes with anticoagulants (K2EDTA, heparin). The plasma was stored at −70 °C until needed.

#### 2.3.3. Determination of a Group of Biomarkers of Metabolic Syndrome and Fatigue in Blood Plasma

The determination of the content of biomarkers of metabolic syndrome and fatigue (3-methylhistidine, threonine, creatine, acetyl-L-carnitine, creatinine, uridine, lactic acid, uric acid, 3-hydroxybutyrate, 2-hydroxybutyrate) in blood plasma was performed by HPLC-MS/MS (LCMS-8050 chromatograph mass spectrometer with atmospheric pressure electrospray ionization in combination with a Nexera XR liquid chromatograph) after desalting the samples with acetonitrile. Separation of sample components was performed on a Zorbax SB-C8 150 mm × 4.6 mm × 1.8 μm chromatographic column (Agilent Technologies, Santa Clara, CA, USA), followed by the registration of selected ion transitions with a tandem mass spectrometric detector. Due to the high levels of the biomarkers in biofluids, additional concentration was not required. Biomarkers were identified by retention times and mass-spectrometric characteristics. The software for data management and processing was “LCMS Solution (Version 5.97 SP1)”.

Table S1 presents the metrological characteristics of the procedure for determining the group of biomarkers of metabolic syndrome and fatigue in blood plasma using the HPLC-MS/MS method.

#### 2.3.4. Selective Determination of Non-Esterified Fatty Acids and Esterified Fatty Acids in Blood Plasma

The analysis was performed using gas chromatography–mass spectrometry (GC-MS). Extractive methylation was used to prepare samples for analysis. The mild conditions for sample preparation for GC-MS analysis excluded uncertainties associated with losses or mutual transformations of fatty acids. In this case, the selective determination of NEFAs and EFAs was carried out in one aliquot of the sample. The method for determining the content of NEFAs and EFAs in blood by GC-MS using extractive methylation was proposed in 2015 by Orlova et al. [31] and has been used in a number of studies, including one on the influence of an occupational pathology on the fatty acid profile of the blood [32].

The content of methyl esters of EFAs and NEFAs in blood plasma was measured using a two-stage method for determining methyl esters of fatty acids, given in [31], with partial modification.

The chromatographic separation of the analytes was carried out using a temperature program beginning at 80 °C (1 min), followed by heating to 210 °C (1 min) and then heating to 230 °C (18 min). The carrier gas flow rate through the column was 1 mL/min, the injection mode was splitless—1 min. The temperatures of the ion source and interface were 230 °C and 240 °C, respectively. The delay for the solvent outlet was 9 min. GC-MS analysis was performed in the mode of selected ions characteristic of fatty acid methyl esters. Three ions were scanned for each methyl ester, namely one main and two confirmatory ions, which were subsequently used to identify the detected compounds (Table S2).

The essence of the two-stage method for determining fatty acids was the trans-esterification of acids into the corresponding methyl esters at the first stage (at pH = 11) and the use of the extractive methylation procedure of free acids at the second (at pH = 8). In this case, both procedures were performed with a single 0.1 mL aliquot of the blood plasma sample, sufficient to obtain reliable results while avoiding overloading the chromatography column with sample components.

*Preparation of Extracts*. A 0.1 mL plasma sample was placed in a 15 mL centrifuge tube. Then, 20 μL of each internal standard solution with a concentration of 100 μg/μL was added: palmitic acid D31 (for determining esterified fatty acids) and palmitate methyl ester D31 (for determining free acids).

*Stage 1*. First, 0.5 mL of 0.8 M sodium methylate was added to the resulting plasma solution, and the solution was vortexed for 10 min; then, 2 mL of extractant (hexane) was added, and the mixture was vortexed again for 10 min and then centrifuged for 10 min at 1300 rpm. The supernatant, containing esterified fatty acids, was collected in a chromatographic vial, evaporated to dryness under a stream of nitrogen, redissolved in 200 µL of methylene chloride, and analyzed by GC-MS under the conditions described above.

*Stage 2*. To the remaining lower layer of the sample containing free fatty acids, 3 mL of phosphate-buffered saline, 60 μL of a 2 mg/mL *o*-phosphoric acid solution to achieve pH = 8, 200 μL of tetrabutylammonium hydroxide, 100 μL of methyl iodide (CH3I), and 3 mL of methylene chloride were added. The resulting mixture was vortexed for 10 min and then centrifuged at 1300 rpm for 10 min. The upper aqueous layer was discarded, and the lower organic layer was transferred to a chromatographic vial and evaporated to dryness under a stream of nitrogen; the dry residue was redissolved in 200 μL of methylene chloride and subjected to GC-MS analysis under the conditions described above.

To determine the fatty acid content in blood plasma samples, two ranges of calibration dependences were established: from 0.002 to 200 μg/mL for acids with minor content and, separately, 200 to 2500 μg/mL for acids with high content (palmitic, stearic, oleic, linoleic, arachidonic, and docosahexaenoic). The calibration dependencies were established using the internal standard method, which involved a deuterated methyl ester of palmitic acid. The correlation coefficients for the calibration characteristics corresponded to values of at least 0.9998. The root mean square deviation (RMSD) of the peak area in each range did not exceed 20%. During the validation of the method, the stability of the blood plasma samples was confirmed when stored in a freezer at minus 70 °C for a year and in 2 freeze–thaw cycles. Moreover, during the validation of the method, the characteristics obtained were not inferior to those established by the developers of the method [33]. Across the entire measurement range, the analysis error did not exceed 20%. The standard deviation of the repeatability of the analytical results, σr, was determined from a series of parallel acid measurements in the same plasma sample (5 series, *n* = 3). The values did not exceed 15%, while the relative error of the analytical results did not exceed 25%.

#### 2.3.5. Biochemical Analysis

Biochemical parameters were determined on a Sapphire 400 analyzer using commercial RANDOX kits. Methods for determining the concentration of malondialdehyde and activity of esterases are described in [34,35,36]. The activity of each sample was measured in triplicate, and the coefficient of variation was 1.8%.

### 2.4. Statistical Data Processing

Our study was largely exploratory in nature and was limited to a small cohort of patients with occupational pathology residing in Novocheboksarsk and registered with Medical and Sanitary Unit No. 29. For these and a number of less significant reasons, we were unable to predict the effects of parameters such as free and esterified fatty acid concentrations and butyrylcholinesterase and albumin esterase activity. For the baseline biochemical parameters, the desired sample power was 200 individuals (effect size 0.4, significance level 0.05, power 0.8). A total of 202 former employees were invited for the examination. It was assumed that approximately 50% would be in the OP group and the rest in the control group. After collecting their medical histories, 49 individuals were excluded from the study because they had occupational diseases not associated with organophosphate exposure or contact with other chemical compounds, which prevented their inclusion in the control group. Thus, the acceptance rate was 76%. Another 10 people did not attend the test due to personal circumstances, and blood samples were not collected from them.

Statistical data processing was carried out using the GraphPad Prism 8.4.3 program. For descriptive statistics, medians with ranges from minimum to maximum and interquartile ranges were used. Testing for normality of distribution was carried out in several ways: the D’Agostino–Pearson omnibus normality test, Shapiro–Wilk normality test, and Kolmogorov–Smirnov normality test. To compare two groups of unrelated data, the Mann–Whitney test was used, and Fisher’s exact test was used to compare the qualitative (nominal) characteristics in two independent samples. Spearman’s rank correlation coefficient was used to measure the relationships between two variables. ROC analysis was used to calculate the area under the curve. An online resource (https://www.ai-therapy.com/psychology-statistics/power-calculator, accessed on 2 August 2025) was used to calculate the post hoc power.

## 3. Results

### 3.1. Sociodemographic Data and Concomitant Diseases

The characteristics of the groups of elderly patients are presented in Table 1. The formed groups did not differ in average age and gender composition. The BMI was calculated using the formula I = m/h^2^, where m is body weight in kg and h is height in m. According to the WHO recommendations, depending on the BMI value, a distinction is made between underweight, a normal body weight, overweight or pre-obesity, and obesity of the first, second, or third degrees. To compare the BMIs of those in the control group and the group of OP patients, the Mann–Whitney test was used. Statistically significant differences were found between the groups, but these differences had no clinical significance; the average values in both groups were in the overweight range. The proportions of people with a normal weight, overweight, and obesity in each group are presented in Table 1 for reference. For correct statistical processing, obesity of the second and third degrees was combined into one category. Smokers included all those who had ever smoked, regardless of the intensity and duration of smoking. In the OP group, the proportion of smokers was four times higher (*p* < 0.05). In both groups, employees with secondary specialized education predominated.

**Figure 1:**
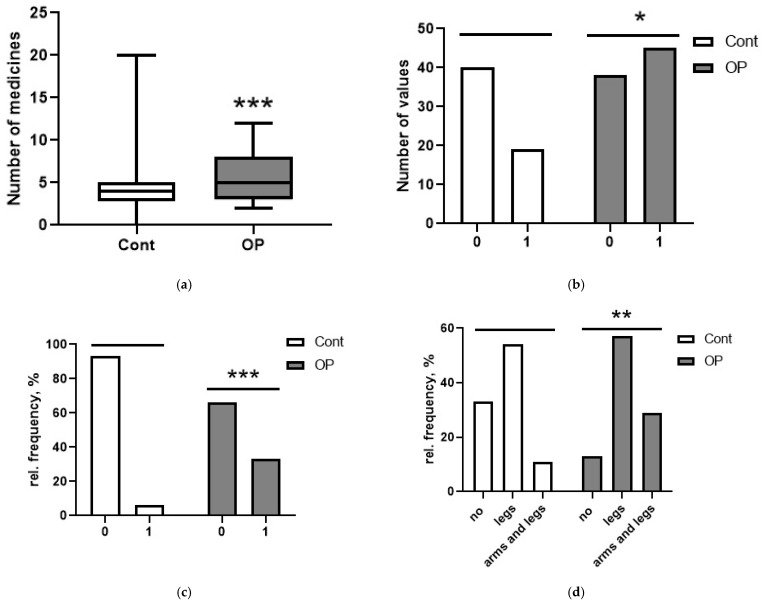
(**a**) Drug load; (**b**) history of gastrointestinal diseases (0—no disease, 1—gastrointestinal diseases); (**c**) history of hepatitis (0—no diagnosis, 1—hepatitis); (**d**) frequency of polyneuropathy diagnosis depending on localization. *, **, ***—differences from controls are statistically significant (*p* < 0.05, *p* < 0.01, *p* < 0.001).

The medications frequently or constantly taken by the respondents were also recorded. To quantify this influencing factor, a general term, “drug load”, was introduced, which was characterized by the number of named medications regardless of their group (Figure 1a). Half of the patients in the control group took three to five medications daily, and patients with occupational diseases took three to eight, i.e., their drug load was higher (*p* < 0.001). The following diagnoses were noted from the medical histories of the subjects: ischemic heart disease; hypertension; gastrointestinal diseases, including hepatitis (of toxic or other genesis); diabetes mellitus; musculoskeletal system diseases; episodes of acute cerebrovascular accident or chronic cerebrovascular disease. Diseases of all listed groups, with the exception of gastrointestinal diseases, occurred in the subjects in the control group and OP patients with comparable frequency. However, gastrointestinal diseases (especially hepatitis) were significantly more common in the OP group (Figure 1b,c). Polyneuropathy was diagnosed during the examination. In the control group, 39 people were diagnosed with this condition, which was 66% of the total number of patients in the group. In the OP group, polyneuropathy was diagnosed in 87% of the patients (*p* < 0.01). It should be noted that approximately 20% of patients in each group may have had polyneuropathy of diabetic origin. Polyneuropathy of the lower extremities was diagnosed more often, but only 12% of the control group had polyneuropathy of the upper and lower extremities, while, in the OP group, there were 29% of such patients (*p* < 0.05). Figure 1d shows the polyneuropathy distribution: in the OP group, polyneuropathy in a more severe form (of the upper and lower extremities) was more common.

To assess cognitive impairment, the subjects were asked to take three tests and also to independently assess their well-being according to several parameters. Self-assessment considered problems with balance and difficulty performing everyday activities (answer options: yes/no); problems with memory and anxiety (answer options: yes/no/sometimes); and personality changes (answer options: yes, no, difficult to answer).

Subsequently, for the statistical assessment of problems with memory and thinking, patients were regrouped into two categories—negative answers and positive ones (yes or sometimes). Answers about anxiety were also combined into two similar categories. During further processing, only strictly negative or positive answers were retained among the answers about personality changes (therefore, the number of respondents decreased). The results of the self-assessment of well-being before and after grouping are presented in Table 2.

When analyzing the questionnaire for the assessment of patients’ well-being, problems with memory and thinking were noted by the majority of respondents from the control and OP groups (87% and 95%, respectively). In the control group, 69% of respondents had difficulty maintaining balance, while, in the OP group, this figure was 84% (*p* < 0.001). At least sometimes, 87% of respondents experienced anxiety, melancholy, and depression, regardless of the group. More than half of the respondents in both groups noted changes in their personality. Difficulties in performing everyday activities due to memory problems were experienced by 48% of respondents in the control group and a quarter more (76%) in the OP group (*p* < 0.0001). No differences were found between the groups when assessing cognitive function using the Mini Mental State Examination (MMSE), Self-Administered Gerocognitive Exam (SAGE), and “Clock” test; the results are presented in Table 3.

When assessing the neurological status, subjective and objective symptoms were assessed. Subjective symptoms included complaints of increased blood pressure, headache, dizziness, back and joint pain, weakness and fatigue, paresthesia or numbness of the extremities, vasomotor reactions, and psychoemotional disorders (mainly associated with sleep disturbances). One point was added for each of the listed symptoms; thus, subjective symptoms were assessed on a 10-point scale (0–9 points). The results are presented in Table 4. In the OP group, respondents noted at least three subjective symptoms from the list, and those in the control group noted at least one symptom. Although the medians in both groups were equal, we observed more pronounced symptoms in the OP group (*p* < 0.05). Among the objective symptoms, the presence of pathological foot and hand reflexes was taken into account. In both groups, the presence of pathological foot reflexes was noted in 7% of patients. The presence of pathological wrist reflexes was detected in 12% of the examined patients in the OP group and only in one person (2%) in the control group (*p* < 0.05).

Coordination disorders (intention, ataxia, missed aim) and craniocerebral changes (nystagmoid, nystagmus, asymmetry of the nasolabial fold, weakness of convergence, deviation of the tongue, symptoms of oral automatism) were assessed using a scoring system (one point for each of the detected symptoms).

In both groups, on average, two signs of coordination disorders and the same number of signs of craniocerebral changes were noted; however, due to differences in maximum values and the third quartile, craniocerebral changes were more common in the OP group (*p* < 0.001).

Vibration sensitivity disorders were detected in 94% of patients in the OP group, which was higher than in the controls, where such disorders were found in 80% of the examined patients. Impaired distal sensitivity was detected in 56% of those examined in the control group. In the OP group, such impairments were found in 83% of those examined (*p* < 0.001). Moreover, during the examination, inhibition (up to complete absence) of abdominal, Achilles, and plantar reflexes was noted (Table 4). Hypothermia of the extremities in both groups occurred in 6–7% of those examined and hyperhidrosis in 38–44%.

### 3.2. Biochemical Parameters

Depending on the volume/quality of the samples obtained (in some elderly people, it is extremely difficult to take an adequate amount of blood), the maximum possible number of parameters was measured. For each parameter, the number of measurements is indicated in the bottom row (Table S3). Of the 34 basic biochemical parameters determined with the biochemical analyzer, statistically significant changes were found for only 11, with their median values not exceeding the reference range (clinical norm) and the differences being due to significant individual deviations.

**Figure 2:**
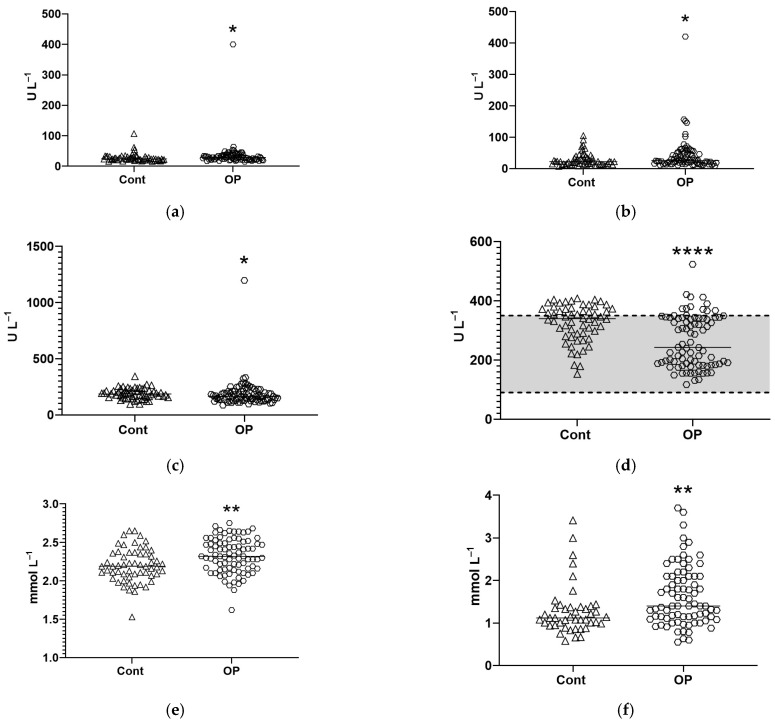

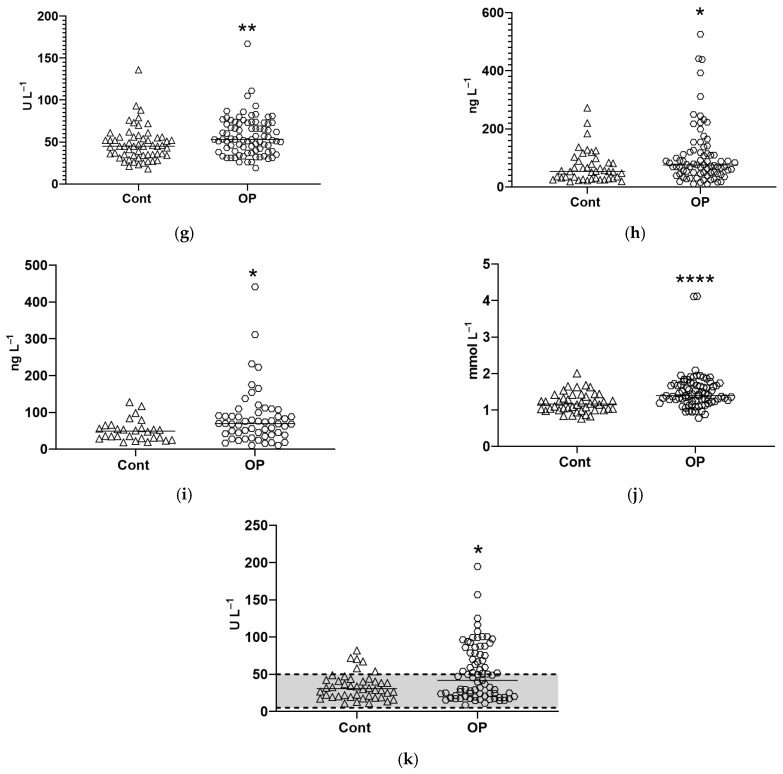
Biochemical parameters measured on the Sapphire 400 biochemical analyzer (statistically significant changes are shown): AST activity (**a**), GGT activity (**b**), ALP activity (**c**), LDH activity (**d**) (the dotted line indicates the normal range), calcium concentration (**e**), HDL concentration (**f**), amylase activity (**g**), ferritin concentration without separation by gender (**h**), ferritin concentration in the blood of women (**i**), TAS (**j**), lipase activity (**k**) (normal range is depicted by dotted lines). *, **, ****—differences from the control group (Mann–Whitney test) are statistically significant, *p* < 0.05, *p* < 0.01, *p* < 0.0001, respectively.

Of the 11 parameters with statistically significant differences, three may be associated with disorders in endothelial cell condition: aspartate aminotransferase (AST), gamma-glutamyltransferase (GGT), and alkaline phosphatase (ALP) (the last two indicate the condition of the blood–brain barrier and endothelial cells) [22]. The average values of AST and GGT activity in the OP group were slightly higher than in the controls, while the statistical significance of these changes was achieved mainly due to high values in individual patients (Figure 2a,b). ALP and lactate dehydrogenase (LDH) activity in the OP group was even lower than in the controls. Despite the significance of these changes (*p* < 0.05 and *p* < 0.0001, respectively; Figure 2c,d), it is necessary to note the high individual values, which were present in both groups. The calcium concentration in the OP group was 6% higher than in the control group (*p* < 0.01, Figure 2e). However, the values in both groups were within the normal range, so this difference in itself did not have clinical significance. The median high-density lipoprotein (HDL) concentration values in the OP group were significantly higher (Figure 2f), but clinical significance required a decrease in HDL to less than 1 mmol/L, which was observed in individual patients in both groups. Amylase activity in the OP group was also 18% higher than in the controls, but it lay within the normal range (Figure 2g).

Due to significant differences in reference intervals between patients of different genders, the ferritin concentration was analyzed separately in women and men. The observed increase in its concentration in the OP group was due to an increase in concentration mainly in women (Figure 2h,i).

The median values of the total antioxidant status (TAS) in the OP group were 20% higher than in the controls, while the significance of the differences was achieved due to high values in individual patients (Figure 2j). Lipase activity in the OP group was only 5% higher, but, in this group, the significance of the differences between the groups was due to individual patients with activity that was two to four times higher than normal. To assess these changes, Figure 2k shows individual values, medians, and a normal range rather than box plots.

**Figure 3:**
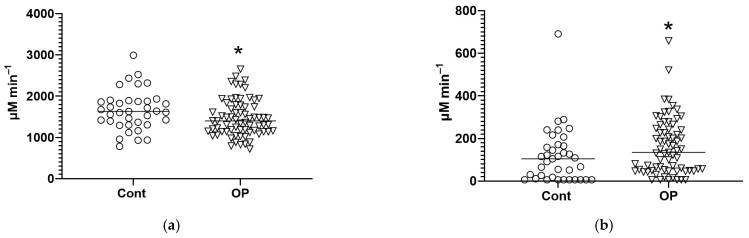
Butyrylcholinesterase activity by acetylthiocholine (**a**) and albumin esterase activity (**b**). *—differences from the control group (Mann–Whitney test) are statistically significant, *p* < 0.05.

An additional study of the esterase profiles of patients was performed in “manual” mode on a plate reader (Table S4). We found a decrease in the activity of butyrylcholinesterase (BChE) by acetylthiocholine (ATCh) as a substrate in the OP group compared to the controls (Figure 3a), but the most unexpected finding was an increase in the esterase activity of albumin in the OP group of 29% (Figure 3b).

Given that palmitate inhibits albumin esterase activity [36,37], the effects of other major fatty acids on this activity are of great interest. We conducted a correlation analysis to search for a possible relationship between this activity and the levels of the following major free (non-esterified) fatty acids: palmitic, stearic, oleic, linoleic, arachidonic, and docosahexaenoic (Table 5).

High correlation levels (0.7 and higher) were not revealed. However, statistically significant relationships were found for the selected NEFAs. When considering the array separately, which included only the subjects from the OP group, the strength and significance of the relationships revealed increases. The most significant and close relationships were found between albumin esterase activity and the arachidonic acid concentration. When considering the subjects from the control group, statistically significant correlations were not revealed. The correlation analysis of all six fatty acids, as well as the sum of arachidonic and docosahexaenoic acids, did not reveal an increase compared to arachidonic acid alone.

**Figure 4:**
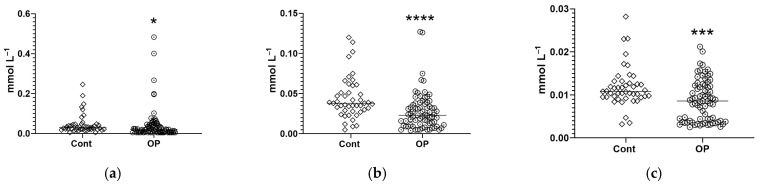
Concentrations of 3-hydroxybutyrate (**a**), 2-hydroxybutyrate (**b**), and acetyl-L-carnitine (**c**) in the blood plasma of the control group (Cont) and patients with occupational pathology (OP). *, ***, ****—differences from the control group (Mann–Whitney test) are statistically significant, *p* < 0.05, *p* < 0.001, *p* < 0.0001, respectively.

Additional metabolic biomarkers (Table S5, Figure 4a–c) were determined using chromatography–mass spectrometry equipment. In the OP group, a 1.4-fold decrease in the concentration of 3-hydroxybutyrate was found compared to the controls. The decrease in the concentration of 2-hydroxybutyrate was more pronounced—1.6-fold (*p* < 0.0001). 3-Hydroxybutyrate is a ketone body, and its increased concentration in the blood causes ketosis. As an intermediate product of fatty acid oxidation, it accumulates in the bodies of patients with diabetes, being, in turn, a precursor of acetoacetate. At the same time, 3-hydroxybutyrate can be used as an energy source for the brain when there is a low level of glucose in the blood. 2-Hydroxybutyrate may be useful as an early indicator of insulin resistance in non-diabetic individuals [38]. In the OP group, a 26% (*p* < 0.001) decrease in the acetyl-L-carnitine concentration was found.

In connection with the detected changes in 2-hydroxybutyrate, 3-hydroxybutyrate, and especially acetyl-L-carnitine, the study of the profile of EFAs and NEFAs in the blood plasma is of particular interest. Table S6 lists the names of the studied EFAs and NEFAs by the presence, quantity, and positions of double bonds and Table S7 by the chain length and concentration in the blood plasma; Table S8 presents the concentrations (μg/mL) of EFAs in the blood plasma of the subjects and Table S9 the concentrations of NEFAs.

**Figure 5:**
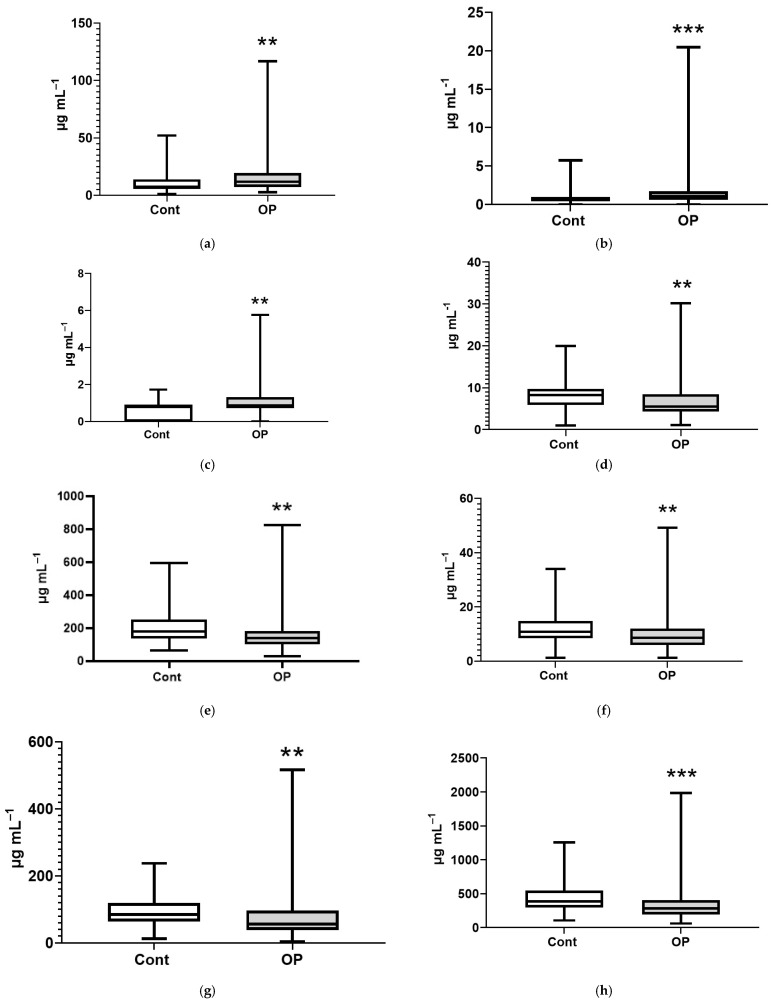

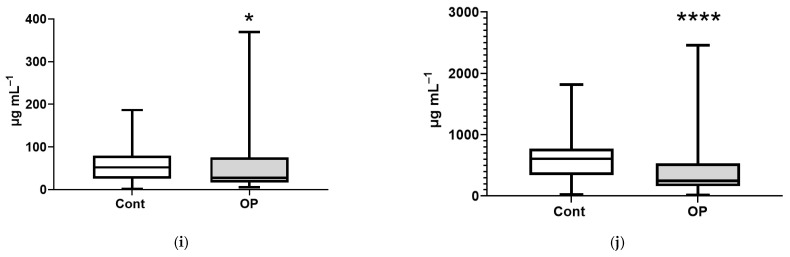
Concentrations (μg/mL) of EFAs in the blood plasma of subjects: myristic C14:0 (**a**), myristoleic C14:1 (**b**), erucic C22:1n9 (**c**), margaric C17:0 (**d**), stearic C18:0 (**e**), eicosadienoic C20:2 (**f**), eicosatrienoic C20:3n8 (**g**), arachidonic C20:4n6 (**h**), eicosapentaenoic C20:5n3 (**i**), docosahexaenoic C22:6n3 (**j**). *, **, ***, ****—differences from the control group (Mann–Whitney test) are statistically significant, *p* < 0.05, *p* < 0.01, *p* < 0.001, *p* < 0.0001, respectively.

Note the statistically significant changes in the concentrations of EFAs in the OP group relative to the controls. An increase in the concentrations of myristic and myristoleic acid esters was revealed, reaching 1.6- and 1.7-fold, respectively (Figure 5a,b), and erucic acid by 11% (Figure 5c). A decrease in concentration was shown for esters of margaric, stearic, and eicosadienoic acids, reaching 1.5-, 1.3-, and 1.25-fold, respectively (Figure 5d–f); for eicosatrienoic acid, this was 1.5-fold (Figure 5g); for arachidonic acid, this was 1.4-fold (Figure 5h); for eicosapentaenoic acid, this was 1.9-fold (Figure 5i); and for docosahexaenoic acid, this was 2.5-fold (Figure 5j).

**Figure 6:**
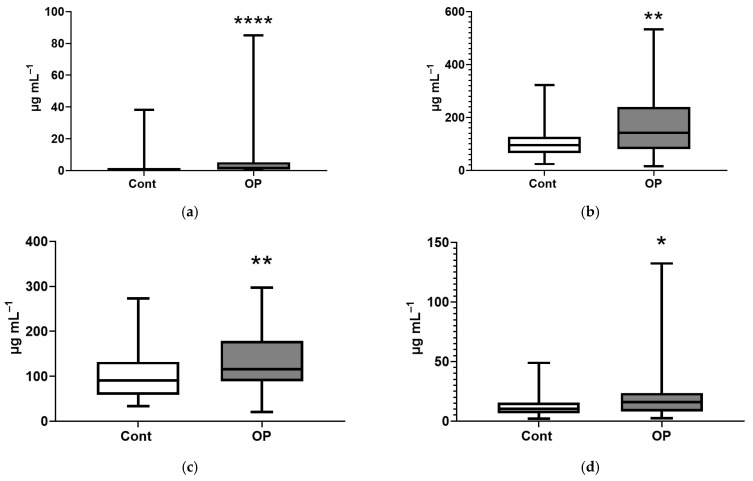

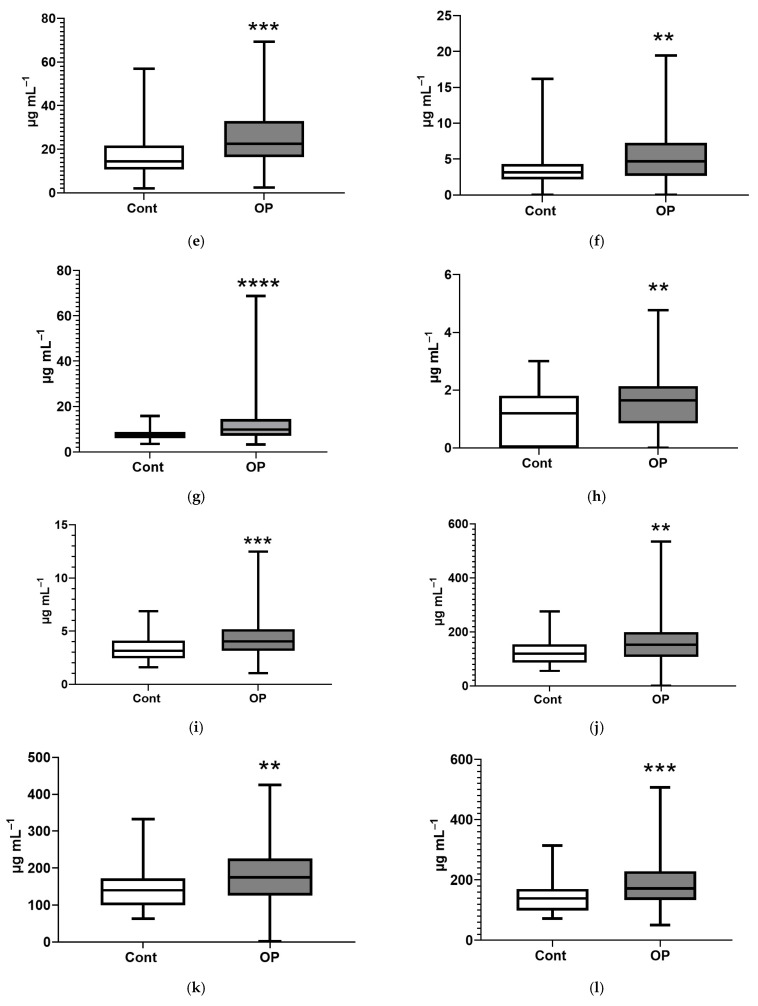

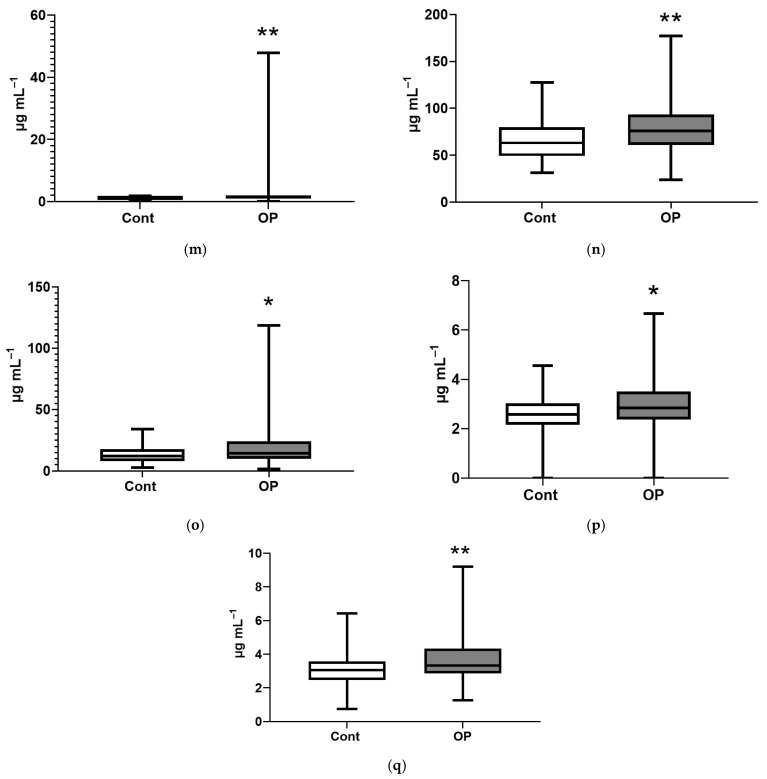
Concentrations (μg/mL) of NEFAs in the blood plasma of subjects: heptadecenoic C17:1 (**a**), docosahexaenoic C22:6n3 (**b**), arachidonic C20:4n6 (**c**), eicosapentaenoic C20:5n3 (**d**), eicosatrienoic C20:3n8 (**e**), γ-linolenic C18:3n6 (**f**), myristic C14:0 (**g**), eicosenoic (**h**), eicosadienoic C20:2 (**i**), oleic C18:1n9c (**j**), linoleic C18:2n6c (**k**), palmitic C16:0 (**l**), linoelaidic C18:2t (**m**), stearic C18:0 (**n**), palmitoleic C16:1n7 (**o**), pentadecanoic C15:0 (**p**), margaric C17:0 (**q**). *, **, ***, ****—differences from the control group (Mann–Whitney test) are statistically significant, *p* < 0.05, *p* < 0.01, *p* < 0.001, *p* < 0.0001, respectively.

It is important to note the statistically significant changes in the concentrations of NEFAs in the OP group. An increase in the concentrations of heptadecenoic acid (+70%), eicosapentaenoic (+56%), eicosatrienoic (+55%), docosahexaenoic (+50%), γ-linolenic (+49%), myristic (+38%), eicosenoic (+37%), arachidonic (+30%), eicosadienoic (+28%), oleic (+27%), linoleic (+25%), palmitic (+24%), linoelaidic (+22%), stearic (+20%), palmitoleic (+20%), pentadecanoic (+10%), and margaric (+10%) acids was revealed (Figure 6).

**Figure 7:**
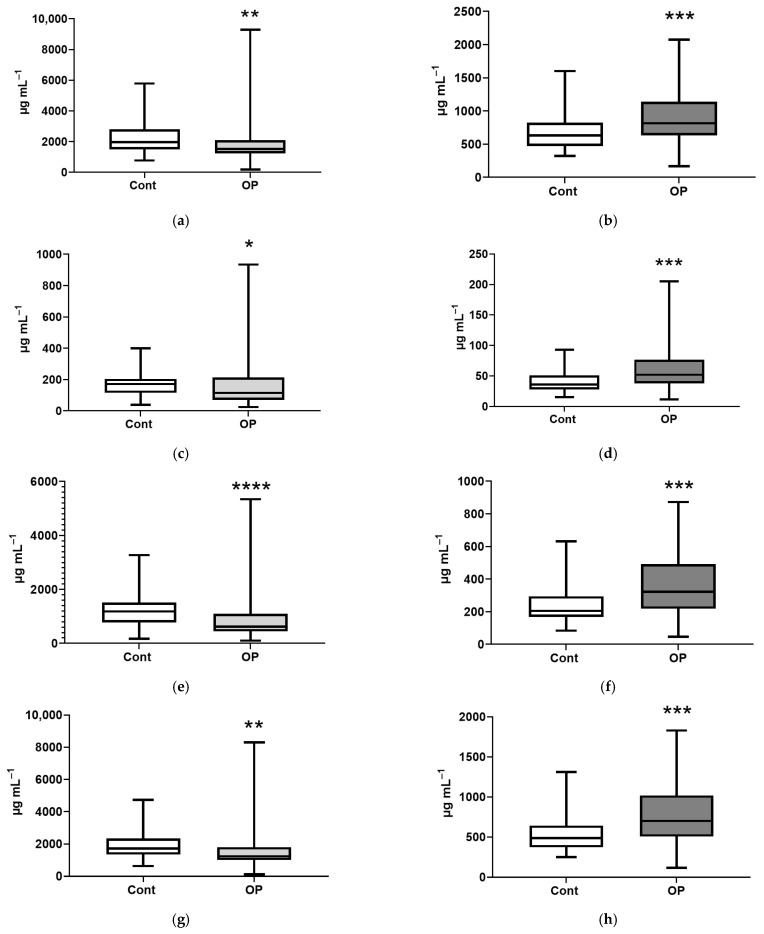

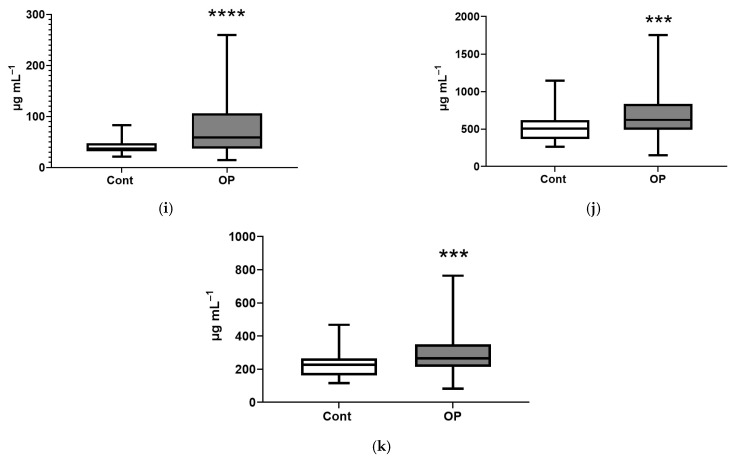
Esterified major fatty acids (**a**), non-esterified major fatty acids (**b**), esterified submajor fatty acids (**c**), non-esterified submajor fatty acids (**d**), esterified ultra-long-chain fatty acids (**e**), non-esterified ultra-long-chain fatty acids (**f**), esterified unsaturated fatty acids (**g**), non-esterified unsaturated fatty acids (**h**), non-esterified minor fatty acids (**i**), non-esterified long-chain fatty acids (**j**), non-esterified saturated fatty acids (**k**). *, **, ***, ****—differences from the control group (Mann–Whitney test) are statistically significant, *p* < 0.05, *p* < 0.01, *p* < 0.001, *p* < 0.0001, respectively. Note: boxes for control group indicators have a white background; boxes for OP group indicators that are reduced relative to the control have a light gray background; boxes for indicators that are increased relative to the control have a dark gray background.

For a more detailed analysis of the obtained data, we grouped EFAs and NEFAs as follows: major, submajor, minor, long-chain (C13-C21), ultra-long-chain (C22 and longer), saturated, unsaturated, and unsaturated ratios n3/n6, n3/(n6 + n9), and n3/(all fatty acids) (Table S10). In the OP group, a decrease in the amount of esterified major fatty acids of 23% was observed, with a simultaneous increase in the amount of free major fatty acids of 30% (Figure 7a,b). A similar picture was observed for the amounts of submajor fatty acids—a decrease in esterified of 32% and an increase in non-esterified of 44% (Figure 7c,d). The amount of esterified ultra-long-chain fatty acids in the OP group was reduced by 1.5 times compared to the controls, while the amount of free ultra-long-chain fatty acids demonstrated the opposite change (Figure 7e,f). Similar changes were observed when calculating the amounts of unsaturated fatty acids: esterified fatty acids were reduced by 28%, while the amount of non-esterified fatty acids was increased by an average of 44% (Figure 7g,h). In addition, in the OP group, the amounts of non-esterified minor, long-chain, and saturated fatty acids were increased—by 58%, 23%, and 17%, respectively (Figure 7i–k).

**Figure 8:**
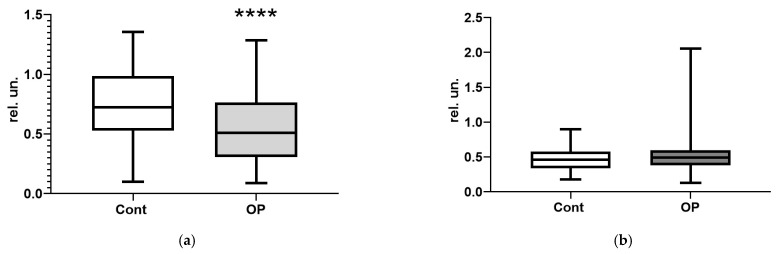

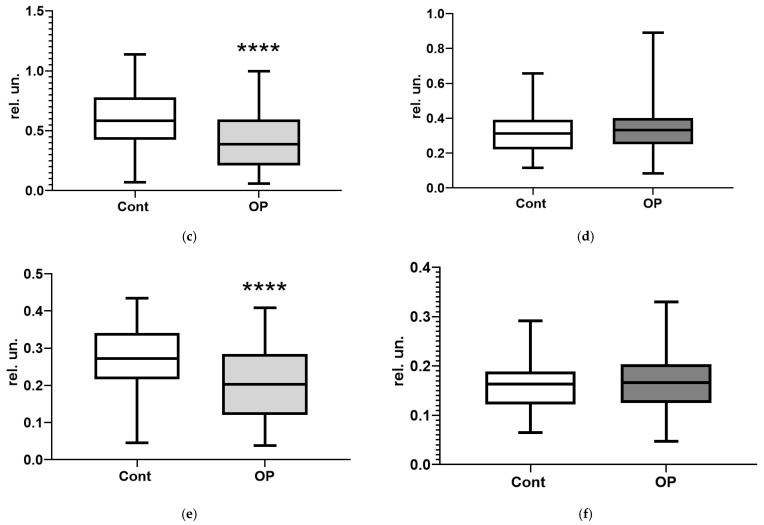
Ratios of omega-3 to other groups of unsaturated fatty acids: esterified n3/n6 (**a**), non-esterified n3/n6 (**b**), esterified n3/(n6 + n9) (**c**), non-esterified n3/(n6 + n9) (**d**), esterified n3/others (**e**), non-esterified n3/others (**f**). ****—differences from the control group (Mann–Whitney test) are statistically significant, *p* < 0.0001.

Changes in the ratios of omega-3 to other unsaturated fatty acids were observed only for the esterified forms. All three calculated indices indicated a significant (*p* < 0.0001) decrease in the concentration of esterified omega-3 fatty acids in the OP group compared to the controls. At the same time, the balance of free fatty acids did not change (Figure 8a–f).

Table 6 includes only statistically significant differences between the OP group and the controls. It is noteworthy that there was a marked increase in the concentrations of esterified myristoleic acid and myristic acid (both esterified and free forms). Chronic myristic acid supplementation has been shown to worsen obesity-associated insulin resistance, and this effect is partly mediated by increased adipose tissue inflammation and increased resistin secretion [39]. At the same time, it should be noted that high myristate levels may be necessary to meet the urgent energy needs of newborns [40].

The most pronounced relative increase compared to the controls was observed for heptadecenoic and eicosapentaenoic acids, reaching 1.7- and 1.5-fold, respectively. Consistent changes in concentration were observed for the sums of major, submajor, ultra-long-chain, and unsaturated fatty acids—the concentration of esterified fatty acids was reduced, while non-esterified fatty acids were increased. At the same time, the sums of free minor (probably due to the concentrations of heptadecenoic, γ-linolenic, and eicosenoic), long-chain, and saturated fatty acids were increased.

**Figure 9:**
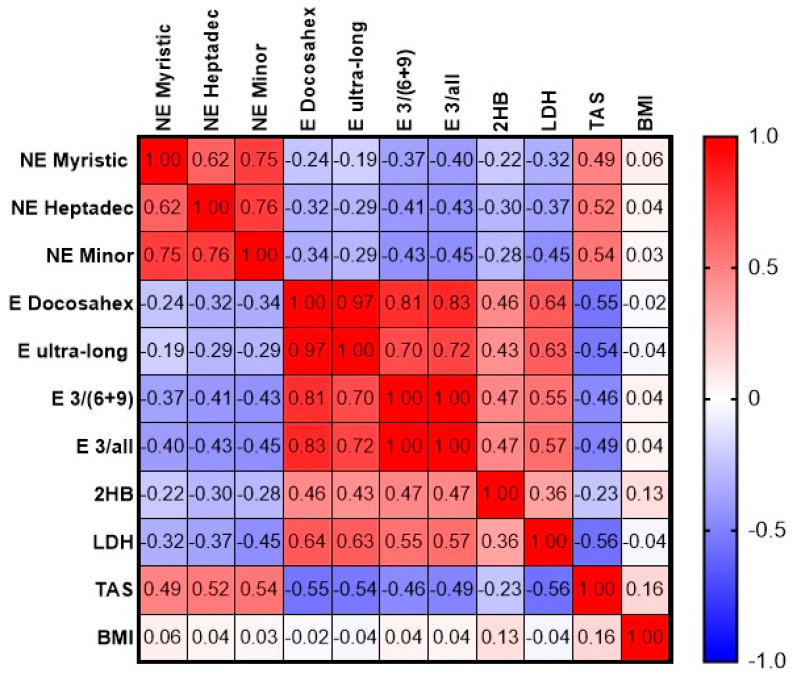
Correlation matrix of the most reliable indicators (entire array). Abbreviations: E—esterified, NE—non-esterified, Docosahex—docosahexaenoic acid, ultra-long—ultra-long-chain fatty acids, 3/(6+9)—(omega-3 FAs)/(omega-6 FAs + omega-9 FAs), 3/all—omega-3 FAs/all FAs), 2HB—2-hydroxybutyrate, LDH—lactate dehydrogenase, TAS—total antioxidant status, BMI—body mass index.

**Figure 10:**
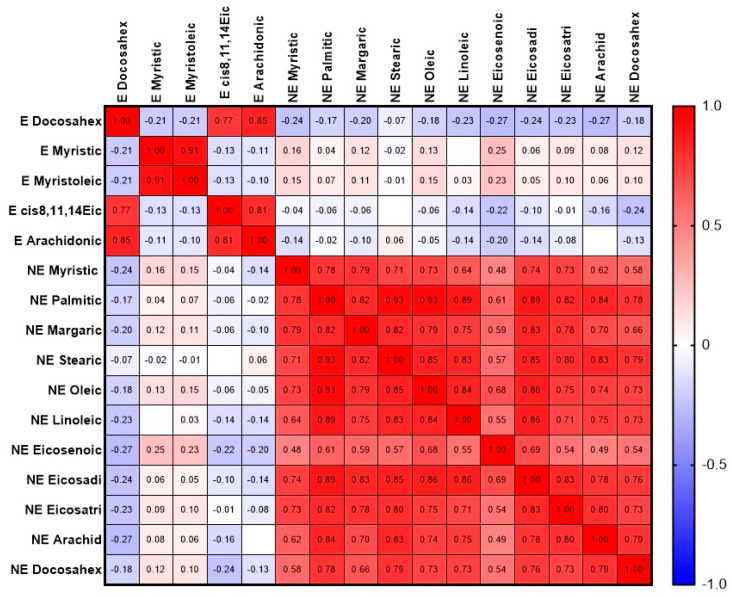
Correlation matrix of fatty acids, showing statistically significant differences between groups. Abbreviations: E—esterified, NE—non-esterified, cis8,11,14Eic—cis-8,11,14-eicosatrienoic, Docosahex—docosahexaenoic acid, Arachid—arachidonic, Eicosadi—eicosadienoic, Eicosatri—eicosatrienoic.

At the next stage, we examined how the most reliable indicators correlated with each other (Figure 9) and all statistically significant ones with the most important ones from the first correlation matrix (Figure 10).

If we exclude the obvious correlation links (the inclusion of myristic and heptadecenoic free fatty acids in the total amount of minor free fatty acids, as well as esterified docosahexaenoic among esterified ultra-long chain acids and the indices n3/(n6 + n9) and n3/all FAs), then five correlation dependencies, presented in Table S11, remain for further consideration. The esterified docosahexaenoic acid–LDH and LDH–TAS correlations are stronger in the OP group compared to the correlations in the overall sample, and they lose their strength and statistical significance in the control group. The non-esterified heptadecenoic–TAS pair demonstrates a weak but statistically significant correlation in all three samples, with minimal interaction observed in the control group. The non-esterified myristic–heptadecenoic and esterified docosahexaenoic–TAS pairs demonstrate the weakening of the correlation in the OP group, without loss of statistical significance (likely due to the reduced number of samples). We next exclude non-esterified myristic and heptadecenoic fatty acids from further consideration as minor components and examine the correlations between esterified docosahexaenoic fatty acid, LDH activity, and the TAS concentration and other statistically significant parameters (Table S12).

All three parameters examined in the OP group have a moderate correlation with the calcium concentration (negative for esterified docosahexaenoic acid and LDH and positive for TAS). However, in the control group, this correlation is significantly weakened or even absent (with LDH). The LDH–calcium correlation pair is also interesting in that the confidence intervals calculated for the correlation coefficients do not overlap, indicating the reliability of the obtained pattern—a moderate negative correlation in one group and complete absence in the other. A similar pattern is seen for the LDH–ALP pair: in the OP group, there is a moderate positive correlation between the parameters, while, in the control group, it is negligible.

Esterified docosahexaenoic acid in the OP group also demonstrated strong positive correlations with other esterified fatty acids—margaric, eicosadienoic, cis-8,11,14-eicosatrienoic, arachidonic, and eicosapentaenoic. However, these correlations were not significantly weakened in the control group. The next step in the data analysis was to search for possible relationships between other statistically significant parameters. The results are presented in Figure 10 as a correlation matrix. We also examined correlations separately in the control group and the OP group (results are presented in Table S13).

In the overall dataset, as well as in the control and OP groups separately, NEFAs correlated with NEFAs, and EFAs correlated with EFAs. Among EFAs, a correlation was found between the minor fatty acids myristic and myristoleic, as well as a strong positive correlation between arachidonic acid and docosahexaenic and cis-8,11,14-eicosatrienoic acids. These correlations were comparable in strength across all groups. Among NEFAs, 23 correlation pairs with strong relationships were found, with the majority of the components in these pairs belonging to major fatty acids. Based on the change in correlation strength across the datasets, two correlation pairs of free fatty acids are of interest: linoleic–palmitic and eicosenoic–eicosadienoic. The first pair had the highest correlation coefficient (0.95) in the control group, while, in the OP group, it decreased to 0.84; the confidence intervals for these coefficients did not overlap. For the second pair, we observed the maximum correlation coefficient (0.82) in the OP group, while, in the control group, it decreased to 0.45. The reliability of this pattern is confirmed by the non-overlapping confidence intervals.

## 4. Discussion

In one of our recent studies, we proposed that OP-induced pathology loses its specificity over time and essentially acts as a trigger for aging and age-related diseases [10]. In addition to the effect on cholinesterases, organophosphates and some products of their destruction have an inhibitory effect on carboxylesterases of intestinal enterocytes, hepatocytes, and endothelial cells, which significantly affects homeostasis and determines the pathogenesis of cardiovascular and neurodegenerative diseases, accelerating the aging of the body [21,22]. Primary changes cause secondary changes—for example, at the level of epigenetic modifications, changes in the balance of the intestinal microbiota, etc.—forming a vicious circle. Identifying a toxic trace in the spectrum of age-related diseases is an extremely complex task. However, with the advancement of modern technological platforms and methodological research algorithms, this task is far from impossible. We have attempted to approach a solution to this problem. This paper presents the results of a diagnostic examination of a unique cohort of patients who were involved in the production of toxic chemicals during the 1980s. The medical histories of the examined individuals in both groups (OP and control) included common age-related diseases: ischemic heart disease, hypertension, gastrointestinal diseases, diabetes mellitus, musculoskeletal system diseases, cerebrovascular diseases, and signs of metabolic syndrome. Notably, the OP group exhibited a significantly higher prevalence of hepatitis; polyneuropathy affecting the upper and lower extremities; pathological hand reflexes; and disturbances of vibration and distal sensitivity.

Among the biochemical parameters, the decrease in BChE activity with ATCh as a substrate observed in the OP group seems important, as it may indicate the disrupted synthesis and/or post-translational modification of the enzyme in the liver. This phenomenon is observed during chemotherapy and various diseases—neurodegenerative, cardiovascular, infectious, and oncological conditions [41,42,43,44,45,46,47].

An intriguing finding was the increase in albumin esterase activity in the OP group, despite similar albumin concentrations in both groups. We emphasize that we refer to the genuine esterase activity of albumin, primarily involving the Sudlow I binding site [36,37]. The literature indicates that several different factors influence albumin’s ligand-binding capacity in relation to fatty acids; notably, these are divalent metal ions such as Cu^2+^, Zn^2+^, and Co^2+^ [48,49,50].

One possible pathway of thrombogenesis mediated by NEFAs is their ability to disrupt the interaction of Zn^2+^ ions with albumin: long-chain NEFAs such as palmitate and stearate alter the conformation of albumin and reduce its ability to bind Zn^2+^, thereby increasing the availability of the ion for the binding and activation of coagulation proteins [49]. Another mechanism of thrombogenesis may be related to the positive correlation that we found between arachidonic acid levels and albumin esterase activity. Platelets incubated with modified albumin were shown to produce significantly more arachidonic acid metabolites and aggregate approximately twice as much as platelets incubated with albumin from healthy subjects [51]. This is explained by the fact that diabetic albumin, as a result of glycoxidation, has an increased amount of free arachidonate available for the formation of active metabolites in platelets. In addition, the binding of NEFAs to albumin or its glycation can affect the reactivity of thiol groups, thereby affecting its antioxidant activity [52].

The observed changes in 3-hydroxybutyrate and 2-hydroxybutyrate in the blood plasma of occupational patients, along with the absence of deviations in glucose levels, suggest that this group of patients does not have pronounced signs of diabetes, which means that signs of peripheral neuropathy are caused by occupational pathology. A decrease in the acetyl-L-carnitine concentration in the OP group was shown for the first time for delayed occupational pathology, but this is a characteristic feature of many other diseases, so acetyl-L-carnitine has even been tested as a potential therapeutic agent [53]. Acetyl-L-carnitine treatment has also been shown to normalize the short-chain fatty acid levels in the gut microbiota, promote intestinal barrier restoration, and reduce proinflammatory cytokine levels (TNF-α, IL-1β) in the brain parenchyma [54].

Our study of a wide range of fatty acids in patients with OP-related pathology and a control group of elderly people revealed a surprisingly consistent pattern of reciprocal relationships between esterified and non-esterified forms of fatty acids: a decrease in the concentration of the former is associated with an increase in the concentration of the latter. Importantly, none of the measured NEFAs showed a statistically significant decrease. This may indicate increased lipase activity [55] and/or decreased uptake and utilization of NEFAs, including by endothelial cells, as observed in obesity [56]. An alternative mechanism for the lipolysis of triglycerides and especially phospholipids with the subsequent uptake of NEFAs is an increase in the expression of endothelial (phospho)lipase [57]. Endothelial lipase is synthesized mainly by endothelial cells, functions on the surfaces of these cells, and exhibits phospholipase A1 activity. Proinflammatory cytokines induce a decrease in plasma HDL-cholesterol concentrations by enhancing endothelial lipase activity, whereas exercise and fish oil, a rich source of docosahexaenoic acid and eicosapentaenoic acids, suppress endothelial lipase activity. Synergistic interaction between endothelial lipase polymorphisms and environmental factors appears to influence coronary heart disease occurrence [58].

Our data provide convincing evidence that NEFAs are a major cytotoxic factor for the vascular endothelium in patients with OP. Multiple studies confirm that NEFAs exert direct cytotoxic effects on endothelial cells and endothelial progenitors, largely through TLR4- and PPARγ-mediated oxidative stress and inflammation [59,60,61,62]. At the same time, some fatty acids have a cytoprotective effect. For example, eicosapentaenoic acid protects against palmitate-induced endothelial dysfunction through the activation of the AMPK/eNOS pathway [63]. An oxidized derivative of docosahexaenoic acid has a cytoprotective effect when exposed to polychlorinated biphenyls on endothelial cells [64]. The probable mechanism of the cytoprotective effect is the activation of NAD(P)H:quinone oxidoreductase, similar to the action of sulforaphane, an Nrf-2 activator, which is abundant in species of the cabbage family [65]. In addition, short-chain fatty acids, which are formed in particular as a result of microbiota metabolism in the gastrointestinal tract, have not only cytotoxic but also cytoprotective effects through the activation of NEFA receptors type 2 and 3, G-protein-coupled receptor 109A, and the inhibition of histone deacetylases [66].

An increased level of polyunsaturated fatty acids, along with an increased omega-3/omega-6 ratio, is a criterion for the effectiveness of dietary supplements and health improvement [67]. Most commercially available docosahexaenoic acid esters are in the form of phospholipids or triglycerides, but glycerophospholipids play a crucial role in transporting docosahexaenoic acid to the brain [68]. The esterified form of docosahexaenoic acid, lysophosphatidylcholine (lysoPC-DHA), is better absorbed by the brain compared to the triacylglycerol form of docosahexaenoic acid. Free docosahexaenoic acid is transported across the outer membrane of the blood–brain barrier using APOE4 receptors, while lysoPC-DHA enters the brain by binding to fatty acid-binding protein 5, which is present in cerebral vascular endothelial cells, and is also transported across the blood–brain barrier using a specific protein, the major facilitator superfamily domain-containing protein symporter 2A (Mfsd2a), which recognizes various lysophospholipids that have choline in their polar heads [69,70,71].

In the OP group, we found significant correlations for esterified docosahexaenoic acid, which were weakened or even absent in the control group: positive with LDH activity (0.67, *p* < 0.0001) and negative with the calcium concentration (−0.66, *p* < 0.0001). Importantly, OP group-specific correlations were also found for LDH–TAS (−0.61, *p* < 0.0001), LDH–calcium (−0.67, *p* < 0.0001), and TAS–calcium (0.63, *p* < 0.0001). These data further support the idea that the bioavailability—and hence the potential beneficial effects—of esterified docosahexaenoic acid is significantly dependent on the type of ester in which it is bound [68]. Esterified docosahexaenoic acid in the OP group also demonstrated strong positive correlations with other esterified fatty acids—margaric (0.70, *p* < 0.0001), eicosadienoic (0.70, *p* < 0.0001), cis-8,11,14-eicosatrienoic (0.77, *p* < 0.0001), arachidonic (0.83, *p* < 0.0001), and eicosapentaenoic (0.78, *p* < 0.0001). However, these correlations were not significantly weakened in the control group.

Organophosphates are the most widely used insecticides worldwide due to their microbial biodegradability; however, this applies not only to soil but also to the human gut, causing intestinal dysfunction and inflammation and increasing the risks of hyperglycemia and diabetes [3,4,72,73,74]. In addition to pesticides and herbicides, organophosphate flame retardants, plasticizers, and lubricants (resorcinol bis(diphenyl)-phosphate, tris(2-chloroethyl) phosphate, triaryl phosphates) are frequently found in biological and global environmental matrices [5,6,75,76], altering the fatty acid spectrum and even the balance of brain neurotransmitters [6].

The mechanisms that explain long-term illness associated with organophosphorus exposure are still under investigation. Both organophosphorus nerve agents and organophosphorus pesticides create covalent adducts via different amino acid residues from a variety of proteins, although choline- and carboxylesterases are their priority targets [77]. Carboxylesterase-1 is expressed not only in the liver but also in vascular endothelial cells; a decrease in its expression and/or activity causes damage to endothelial cells and endothelium-dependent pathology [78]. Diet strongly modulates these effects: a high-fat Western diet enhances organophosphate bioavailability and toxicity, while the Mediterranean diet mitigates them [79]. Some steroid glycosides also modulate the negative effects of organophosphates, reducing macrophage infiltration and proinflammatory cytokine levels but increasing short-chain fatty acid levels [80]. Given the impact of organophosphates on the microbiota and somatic cells of the body, the spectrum of fatty acids and other diagnostic markers deserves close attention and further research, as does the search for means to neutralize the negative effects of organophosphates.

## 5. Limitations

The nature of retrospective studies imposes many limitations. The uniqueness of the sample of patients is closely related to the limitations of the experimental design. The single-center design may limit the external validity, as the sample may not capture the heterogeneity of patients with delayed pathologies after exposure to organophosphates. Moreover, thirty to forty years ago, the biochemical and instrumental analysis capabilities were insufficient for the objective monitoring of patients’ conditions and the frequency and extent of organophosphate-related damage. Another significant limitation in our case was the lack of records of previously deceased individuals with occupational diseases, due to the limited time for which they were stored in the registry. Therefore, we were unable to conduct a Kaplan–Meier or log-rank analysis to estimate survival, which would have strengthened the study. In addition, the absence of a healthy control group that was completely matched in terms of age and socioeconomic status prevented the full isolation of syndrome-specific effects. Many retrospective cohort studies cannot prove causality but only suggest associations, and our study is no exception to this. The main internal contradiction in our retrospective cohort study is likely the fact that changes in the quality of data or exposure status over time can occur, but it was the nature of these changes that was the main focus of our research. A cohort study could establish a temporal relationship, which helps in inferring causality, but the overlapping cause-and-effect relationships that lead to age-related diseases also negate this potential. Confounding variables can affect the results, but, in theory, statistical methods can be used to adjust for them. However, in our case, it was challenging or even impossible to select the most relevant statistical analysis method from the generally accepted ones. Therefore, one of the main goals of further research should be to develop fundamentally new analytical methods using machine learning.

## 6. Conclusions

The anamnesis of the examined patients with OP most often included neurological diseases, hepatitis, and other gastrointestinal diseases, although no differences were found between the groups when assessing cognitive function using the MMSE, SAGE, and “Clock” tests. Of the 34 basic biochemical parameters determined with the biochemical analyzer, statistically significant changes were found for only 11, with their median values not exceeding the reference range. An analysis of some metabolic biomarkers with HPLC-MS/MS revealed, in the OP group a significant decrease in the concentrations of 3-hydroxybutyrate and 2-hydroxybutyrate, which allowed us to exclude the diabetic nature of polyneuropathy and indicated the predominantly OP-induced genesis of this pathology. In the OP group, a significant decrease in the acetyl-L-carnitine concentration was found, which can be one of the most important markers of OP-induced pathology. An additional study of the esterase profiles of the patients revealed a decrease in the activity of BChE, which may indicate a violation of the protein-synthesizing function of the liver, and an increase in the esterase activity of albumin in the OP group. The correlation analysis revealed the most significant relationships between albumin esterase activity and the arachidonic acid concentration in the OP group. A study of a wide range of fatty acids in patients with OP revealed reciprocal relationships between EFAs and NEFAs. Decreases in the ratios of omega-3 to other unsaturated fatty acids were observed only for the esterified forms: by 29% for n3/n6, by 33% for n3/(n6 + n9), and by 26% for n3/(all fatty acids).

Significant correlations for esterified docosahexaenoic acid were found in the patients in the OP group, which were weakened or even absent in the control group: positive with LDH activity and negative with the calcium concentration. Importantly, OP group-specific correlations were also found for LDH–TAS, LDH–calcium, and TAS–calcium. These data further support the idea that the bioavailability—and hence the potential beneficial effects—of esterified docosahexaenoic acid is significantly dependent on the type of ester in which it is bound. Esterified docosahexaenoic acid in the OP group also demonstrated strong positive correlations with other esterified fatty acids—margaric, eicosadienoic, cis-8,11,14-eicosatrienoic, arachidonic, and eicosapentaenoic. However, these correlations were not significantly weakened in the control group.

The data obtained allow us to consider an increased level of NEFAs as one of the main cytotoxic factors for the vascular endothelium, which can determine the specificity of age-related diseases. The modification of albumin properties by arachidonic acid and the decreased bioavailability of docosahexaenoic acid could be molecular links that cause specific manifestations of OP-induced pathology at late stages after exposure. Decreased activity of BChE and the acetyl-L-carnitine concentration are additional markers of liver damage specific to the delayed effects of OP-induced pathology. Epigenetic changes and the contributions of dietary patterns and the intestinal microbiota to the spectra of EFAs and NEFAs require additional research.

## Figures and Tables

**Table 1:** Characteristics of the sample of respondents. BMI is presented as the mean and standard deviation; age and drug load—median, interquartile range, and range from minimum to maximum; %—the proportion of the discussed feature in the sample under consideration.

	Control	OP
**Sociodemographic Data**		
Number of people examined	59	84
Men, *n* (%)	17 (29)	24 (29)
Women, *n* (%)	42 (71)	60 (71)
Age, m ± SD	73 ± 4	74 ± 4
BMI, m ± SD	28.4 ± 4.1	30.0 ± 4.6 *
BMI within normal range (18.5–25), *n* (%)	13 (22.0)	13 (16.1)
Overweight (BMI 25–30), *n* (%)	27 (45.8)	28 (34.5)
Obesity grade 1 (BMI 30–35), *n* (%)	14 (23.7)	29 (35.8)
Obesity grade 2 (BMI 35–40), *n* (%)	4 (6.8)	9 (11.1)
Obesity grade 3, *n* (%) (BMI 40 and more)	1 (1.7)	2 (2.5)
Proportion of smokers, *n* (%)	2 (3.4)	12 (14.6) *
Education (higher), *n* (%)	8 (14.5)	8 (10.4)
Education (secondary specialized), *n* (%)	33 (60.0)	56 (72.7)
Education (secondary), *n* (%)	14 (25.5)	13 (16.9)
Drug load	4 (3; 5) 0–20	5 (3; 8) *** 2–12
**Diseases**		
Ischemic heart disease, *n* (%)	20 (34)	38 (46)
Hypertension, *n* (%)	55 (95)	75 (91)
Gastrointestinal diseases, *n* (%) Including hepatitis, *n* (%)	19 (32) 4 (7)	45 (54) * 27 (33) ***
Diabetes mellitus, *n* (%)	10 (17)	19 (23)
Oncological diseases, *n* (%)	3 (5)	4 (5)
Musculoskeletal system diseases, *n* (%)	54 (92)	78 (95)
Acute cerebrovascular accident or chronic cerebrovascular disease, cerebrovascular disease, *n* (%)	54 (92)	78 (95)
Total with diagnosis of polyneuropathy Including diabetes mellitus, *n* (%)	39 (66)	71 (87) **
	8 (21)	16 (23)
Polyneuropathy of the upper and lower extremities, *n* (%)	7 (12)	24 (29) *

*, **, ***—differences from controls are statistically significant (*p* < 0.05, *p* < 0.01, *p* < 0.001).

**Table 2:** Self-assessment of cognitive impairment among subjects.

	**Response**	**Control**	**OP**
Self-assessment of memory and thinking problems, *n* (%)	Yes	23 (41.8)	45 (56.2)
	Sometimes	25 (45.5)	31 (38.8)
	No	7 (12.7)	4 (5.0)
Self-assessment of balance problems, *n* (%)	Yes	38 (69)	66 (84) *
	No	17 (31)	13 (16)
Self-assessment of feelings of anxiety, melancholy, depression, *n* (%)	Yes	24 (43.6)	45 (56.3)
	Sometimes	25 (43.6)	25 (31.2)
	No	7 (12.8)	10 (12.5)
Self-assessment of personality change, *n* (%)	Yes	28 (50.9)	39 (48.8)
	No	2 (3.6)	8 (10.0)
	Do not know	25 (45.5)	33 (41.2)
Self-assessment of difficulty in performing daily activities, *n* (%)	Yes	27 (49)	59 (75) **
	No	28 (51)	20 (25)
**Data after grouping**			
There are problems with memory and thinking, *n* (%)		48 (87)	76 (95)
There is a feeling of anxiety, melancholy, or depression, *n* (%)		48 (87)	70 (87)
There are personality changes, *n* (%)		28 (53)	39 (54)

*, **—differences from controls are statistically significant (*p* < 0.05, *p* < 0.01).

**Table 3:** Test results.

	Control	OP
MMSE	27 (26; 28) 21–30	27 (26; 28) 13–31
SAGE	16 (12; 18) 6–21	16 (12; 18) 5–21
“Clock” test	9 (8; 10) 4–10	8 (7; 9) 4–10
Total score for three tests	51 (47; 54) 39–60	50 (46; 55) 24–61

**Table 4:** Assessment of neurological status.

	Control (*n* = 59)	OP (*n* = 82)
Subjective symptoms (0 to 9)	8 (6; 9) 1–9	8 (7; 9) * 3–9
Presence of pathological foot reflexes, *n* (%)	4 (7)	6 (7)
Presence of pathological wrist reflexes, *n* (%)	1 (2)	10 (12) *
Impaired coordination (0 to 3 signs)	2 (2; 2) 0–3	2 (2; 3) 1–3
Cranial changes (0 to 6 signs)	2 (1; 2) 0–4	2 (1; 3) *** 0–5
Vibration sensitivity disorder, *n* (%)	47 (80)	77 (94) *
Impaired distal sensitivity, *n* (%)	33 (56)	68 (83) ***
Depression/absence of abdominal reflexes, *n* (%)	39 (66) 15 (25)	55 (67) 21 (26)
Depression/absence of Achilles reflexes, *n* (%)	20 (34) 24 (41)	34 (41) 22 (27)
Depression/absence of plantar reflexes, *n* (%)	28 (47) 21 (36)	30 (37) 30 (37)
Hypothermia of the extremities, *n* (%)	4 (7)	5 (6)
Hyperhidrosis of the extremities, *n* (%)	26 (44)	31 (38)

*, ***—differences from the control group (Mann–Whitney test) are statistically significant, *p* < 0.05, *p* < 0.001, respectively.

**Table 5:** Correlations of albumin activity and the concentrations of some free fatty acids. The results are presented as the r coefficient and 95% confidence intervals.

	Spearman Correlation Coefficient for Full Array, *n* = 104	Spearman Correlation Coefficient for Controls Only, *n* = 36	Spearman Correlation Coefficient for OP Group Only, *n* = 68
Palmitic	0.3842 (0.2014–0.5412) *p* < 0.0001 (****)	−0.0301 (−0.3639–0.3106) *p* = 0.8617	0.4936 (0.2826–0.6590) *p* < 0.0001 (****)
Stearic	0.3488 (0.1619–0.5116) *p* = 0.0003 (***)	−0.0221 (−0.3569–0.3178) *p* = 0.8984	0.4501 (0.2303–0.6261) *p* = 0.0001 (***)
Oleic	0.3281 (0.1390–0.4941) *p* = 0.0003 (***)	−0.0410 (−0.3733–0.3007) *p* = 0.8124	0.4391 (0.2173–0.6178) *p* = 0.0002 (***)
Linoleic	0.3530 (0.1665–0.5151) *p* = 0.0002 (***)	−0.05136 (−0.3822–0.2912) *p* = 0.7661	0.4695 (0.2535–0.6409) *p* < 0.0001 (****)
Arachidonic	0.4956 (0.3299–0.6317) *p* < 0.0001 (****)	0.1300 (−0.2171–0.4478) *p* = 0.4500	0.6364 (0.4635–0.7626) *p* < 0.0001 (****)
Docosahexaenoic	0.46264 (02909–0.6051) *p* < 0.0001 (****)	0.0991 (−0.2467–0.4225) *p* = 0.5653	0.5472 (0.3488–0.06986) *p* < 0.0001 (****)
Sum of arachidonic and docosahexaenoic acids	0.4823 (0.3142–0.6211) *p* < 0.0001 (****)	0.0978 (−0.2479–0.4214) *p* = 0.5704	0.5993 (0.04151–0.7363) *p* < 0.0001 (****)
Sum of six NEFAs	0.4334 (0.2573–0.5816) *p* < 0.0001 (****)	−0.0158 (−0.3515–0.3234) *p* = 0.9270	0.5564 (0.3604–0.7054) *p* < 0.0001 (****)

***, ****—differences from the control group (Mann–Whitney test) are statistically significant, *p* < 0.001, *p* < 0.0001, respectively.

**Table 6:** Main trends in changes in the concentrations of fatty acids in the blood plasma of subjects from the OP group compared to the control group. The table shows the proportion of median values in the control group.

Name of Acid	EFA, %	NEFA, %
Myristic, C14:0	159 **	138 ****
Myristoleic, C14:1	168 ***	
Pentadecanoic, C15:1		110 *
Palmitic, C16:0		124 ***
Palmitoleic, C16:1n-7		120 *
Margaric, C17:0	67 **	110 **
Heptadecenoic, C17:1		171 ****
Stearic, C18:0	78 *	120 **
Oleic, C18:1n9c		127 **
Linoelaidic, C18:2t		122 **
Linoleic, C18:2n6c		125 **
γ-Linolenic, C18:3n6		149 **
Eicosenoic, C20:1		137 **
Eicosadienoic, C20:2	80 **	128 ***
Eicosatrienoic, C20:3n8	66 **	156 ***
Erucic, C22:1n9	112 **	
Arachidonic, C20:4n6	73 ***	128 **
Eicosapentaenoic, C20:5		156 *
Docosahexaenoic, C22:6n3	41 ****	149 **
Sum of major	77 **	130 ***
Sum of submajor	68 *	144 ***
Sum of minor		158 ****
Sum of long chain		123 ***
Sum of extra long chain	52 ****	157 ***
Sum of saturated FAs		117 ***
Sum of unsaturated FAs	72 **	144 ***

*, **, ***, ****—differences from the control group are statistically significant, *p* < 0.5, *p* < 0.01, *p* < 0.001, *p* < 0.0001, respectively.

## Data Availability

The datasets generated and/or analyzed during the current study are not publicly available. However, they are available from the corresponding author upon reasonable request.

## Supplementary Materials

The following supporting information can be downloaded at: https://www.mdpi.com/article/10.3390/diagnostics15243246/s1, Table S1: Metrological characteristics of the procedure for determining functional state markers in blood plasma by HPLC-MS/MS; Table S2: Characteristic ions of the determined fatty acid methyl esters; Table S3: Biochemistry (biochemical analyzer); Table S4: Biochemistry (plate reader); Table S5: Results of chemical analysis with HPLC-MS/MS of biomarkers in the blood of patients; Table S6: Characteristics of FA by the presence, quantity and position of double bonds; Table S7: Characteristics of fatty acids depending on chain length and concentration in blood plasma; Table S8: Concentrations (μg/mL) of EFA in the blood plasma of the patients; Table S9: Concentrations (μg/mL) of NEFA in the blood plasma of the subjects; Table S10: Concentrations (μg/mL) of the main classes of esterified and non-esterified fatty acids in the blood plasma of the patients; Table S11: Correlation pairs of the most reliable statistically significant indicators; Table S12: Correlation pairs of esterified docosahexaenoic fatty acid, LDH activity and TAS concentration with other statistically significant parameters; Table S13: Correlation pairs of other statistically significant indicators.

## Abbreviations

ALP	alkaline phosphatase
ALT	alanine aminotransferase
AST	aspartate aminotransferase
ATCh	acetylthiocholine
BChE	butyrylcholinesterase
BMI	body mass index
BTCh	butyrylthiocholine
EFA	esterified fatty acid
FA	fatty acid
GGT	gamma-glutamyltransferase
HDL	high-density lipoprotein
LDH	lactate dehydrogenase
MDA	malondialdehyde
MMSE	Mini Mental State Examination
NEFA	non-esterified fatty acid
OP	occupational pathology
PON1	paraoxonase-1
PUFA	polyunsaturated fatty acid
RMSD	root mean square deviation
ROS	reactive oxygen species
SAGE	Self-Administered Gerocognitive Exam
SCFA	short-chain fatty acid
SCSFA	short-chain saturated fatty acid
SFA	saturated fatty acid
TAS	total antioxidant status
VLDL	very low-density lipoprotein
