# Supplementary material for: Characteristics of the Fatty Acid Composition in Elderly Patients with Occupational Pathology from Organophosphate Exposure
Source: Diagnostics (Basel). 2025 Dec 18;15(24):3246. doi: 10.3390/diagnostics15243246 (PMC12731968; doi:10.3390/diagnostics15243246)
Supplement: Supplementary file 1 [file diagnostics-15-03246-s001.zip › diagnostics-3993822-supplementary.pdf]

Supplement

Table S1 – Metrological characteristics of the procedure for determining functional state markers in blood plasma by HPLC-MS/MS

Biomarker	Measurement range (μg/cm³)	Repeatability index (relative standard deviation of repeatability), σ_r, %	Reproducibility index (relative standard deviation of reproducibility), σ_R, %	Trueness index (relative systematic error at probability P=0.95), $\pm\delta_c$, %	Accuracy index (relative error at probability P=0.95), $\pm\delta$, %
3-Methylhistidine	0.005-1	20	22	18	25
Threonine	0.005-1				
Creatine	0.005-1				
Acetylcarnitine	0.005-1				
Creatinine	0.05-10				
Uridine	0.05-10				
Lactate	0.25-50				
Uric acid	0.25-50				
3HB	0.25-50				
2HB	0.25-50				

Note – The values of the procedure accuracy indicator are used when presenting the measurement results issued by the laboratory; assessing the activities of laboratories based on the quality of measurements performed; assessing the possibility of using measurement results when implementing a measurement technique in a specific laboratory.

Table S2 - Characteristic ions of the determined fatty acid methyl esters

##	Methyl ester of acid	Characteristic ions, m/z	The established ratio of ion intensities, %
1	Myristic	**74***,55,143	100:20:10
2	Myristoleic	**55**,74,67	100:55:29
3	Pentadecanoic	**74**,55,143	100:23:9
4	Palmitic D31 (internal standard)	**77**,91,79	100:51:12
5	Pentadecenoic	**55**,74,67	100:49:27
6	Palmitic	**74**,55,143	100:20:9
7	Palmitoleic	**55**,74,67	100:60:34
8	Margaric	**74**,55,143	100:24:10
9	Heptadecenoic	**55**,74,67	100:54:30
10	Stearic	**74**,55,143	100:23:11
11	Elaidic	**55**,74,67	100:56:28
12	Oleic	**55**,74,67	100:61:37
13	Linoleic	**67**,55,79	100:55:34
15	Arachidic	**74**,55,143	100:24:12
16	γ-Linolenic	**79**,67,55	100:99:46
17	Eicosenoic	**55**,74,67	100:49:26
18	α-Linolenic	**79**,55,67	100:70:61
19	Heneicosanoic	**74**,55,143	100:27:13
20	Eicosadienoic	**67**,55,79	100:64:31
21	Behenic	**74**,55,143	100:26:14
22	cis-8,11,14-Eicosatrienoic	**67**,79,55	100:94:55
23	Erucic	**55**,74,67	100:43:33
24	cis-11,14,17-Eicosatrienoic	**79**,67,55	100:75:54
25	Arachidonic	**79**,67,91	100:75:63
26	Tricosanoic	**74**,55,143	100:30:15

27	Docosadienoic	**67**,55,79	100:70:29
28	Lignoceric	**74**,55,143	100:29:15
29	Eicosapentaenoic	**79**,67,91	100:60:57
30	Nervonic	**55**,74,67	100:41:31
31	Docosahexaenoic	**79**,91,67	100:72:64

* - Ions for quantitative determination of analyte content are shown in bold.

Table S3. Biochemistry (biochemical analyzer). Data are presented as median, (interquartile range) and minimum-to-maximum range (2nd row) from minimum to maximum. 3rd row – number of observations.

##	Biomarker	Control	OP	Post-hoc power	AUC
1.	ALT<br>0-50 U/L	19 (16; 26)<br>10-69<br>58	20 (15; 26)<br>7-288<br>84		
2.	AST<br>0-50 U/L	24 (21; 31)<br>16-107<br>58	29 (22; 37) *<br>15-400<br>84	0.229	0.6221
3.	Albumin<br>35-52 g/L	47.0 (44.1; 49.6)<br>36.8-55.0<br>58	46.5 (44.3; 49.5)<br>37.8-63.7<br>84		
4.	Glucose<br>3.5-6.1 mmol/L	5.20 (4.65; 6.10)<br>3.90-15.90<br>57	5.40 (4.73-6.08)<br>3.60-12.21<br>84		
5.	GGT<br>10-55 U/L	22 (14; 34)<br>7-105<br>58	26 (17; 48) *<br>8-420<br>84	0.172	0.6020
6.	Hydroxybutyrate<br>0.03-0.3 mmol/L	0.07 (0.04; 0.15)<br>0.01-1.84<br>44	0.09 (0.06; 0.16)<br>0.01-0.56<br>79		
7.	Total protein<br>66-87 g/L	80.1 (74.7; 83.8)<br>58.3-90.3<br>58	79.8 (75.9; 83.3)<br>63.0-93.3<br>84		
8.	Inorganic phosphate (P_i)<br>0.87-1.45 mmol/L	1.12 (0.99; 1.25)<br>0.63-1.73<br>58	1.12 (0.99; 1.20)<br>0.74-1.48<br>84		
9.	Calcium<br>2.20-2.65 mmol/L	2.19 (2.09; 2.36)<br>1.53-2.65<br>58	2.32 (2.16; 2.51) **<br>1.62-2.75<br>84	0.351	0.6596
10.	Urea<br>2.8-7.2 mmol/L	5.9 (5.3; 7.0)<br>2.9-15.5<br>58	6.0 (5.0; 7.6)<br>3.6-12.8<br>83		
11.	Triglycerides<br>0.1-2.29 mmol/L	1.38 (1.07; 2.05)<br>0.59-4.35<br>58	1.51 (1.12; 2.13)<br>0.47-6.78<br>84		
12.	Creatinine<br>72-127 μmol/L	93.0 (79.0; 110.0)<br>69.0-184.0<br>58	87.5 (78.0; 99.0)<br>50.0-171.0<br>84		
13.	ALP<br>98-279 U/L	187 (156; 219)<br>96-343<br>58	165 (131; 211) *<br>86-1197<br>84	0.167	0.6003
14.	Transferrin<br>2.0-3.8 g/L	2.2 (2.0; 2.4)<br>1.4-3.5<br>43	2.1 (1.9; 2.3)<br>1.4-3.3<br>79		

15.	Bile acids<br>2-10 µmol/L	2.5 (2.0; 4.9)<br>0.5-21.7<br>44	3.3 (2.1; 4.8)<br>1.0-25.5<br>79		
16.	HDL<br>above 0.9 mmol/L	1.12 (0.96; 1.38)<br>0.58-3.41<br>44	1.40 (1.11; 2.10) **<br>0.55-3.70<br>79	0.309	0.6677
17.	LDL<br>below 3.37 mmol/L	3.26 (2.31; 3.66)<br>1.16-5.29<br>44	2.79 (2.25; 3.42)<br>1.40-5.17<br>79		
18.	Creatine kinase<br>24-195 U/L	107 (79; 160)<br>34-1066<br>58	113 (73; 161)<br>37-485<br>84		
19.	Cholesterol<br>0.1-6.19 mmol/L	5.64 (4.53; 6.51)<br>2.46-8.29<br>58	6.00 (4.84; 6.86)<br>3.06-9.49<br>84		
20.	LDH<br>90-350 U/L	340 (287; 374)<br>153-408<br>58	243 (185; 343) ****<br>117-523<br>84	0.579	0.7188
21.	Uric acid<br>202-416 µmol/L	249 (214; 321)<br>147-483<br>58	285 (237; 352)<br>152-543<br>84		
22.	Amylase<br>0-95 U/L	45 (34; 56)<br>18-136<br>58	53 (39; 73) **<br>19-167<br>84	0.271	0.6367
23.	Ferritin<br>(female+male)<br>10-300 ng/mL	53 (29; 89)<br>18-272<br>43	76 (45; 120) *<br>10-526<br>79	0.197	0.6278
24.	Ferritin (women)<br>10-120 ng/mL	49 (30; 63)<br>18-128<br>28	69 (40; 97) *<br>10-441<br>56	0.193	0.6572
25.	Ferritin (men)<br>20-300 ng/mL	89 (27; 137)<br>25-272<br>15	118 (69; 222)<br>30-526<br>23		
26.	TAS<br>1.28-1.83 mmol/L	1.17 (1.01; 1.41)<br>0.77-2.01<br>44	1.40 (1.24; 1.70)<br>****<br>0.78-4.12<br>79	0.532	0.7340
27.	CRP<br>below 5 mg/L	2.6 (1.8; 4.2)<br>0.6-34.4<br>43	2.5 (1.6; 4.4)<br>0.25-73<br>79		
28.	Glycated Hb<br>(HbA1c), 4-6.2%	6.5 (6.1; 7.6)<br>5.3-13.7<br>46	6.7 (6.4; 7.2)<br>5.2-11.9<br>64		
29.	Lipase<br>5.6-51.3 U/L	30.7 (19.6; 42.0)<br>10.9-82.0<br>44	41.7 (20.1; 78.3) *<br>8.7-194.7<br>79	0.175	0.6154
30.	SOD, rel.un./min/mL	6.49 (6.47; 6.53)<br>6.21-6.62<br>39	6.50 (6.47; 6.53)<br>5.58-6.63<br>37		
31.	Fructosamine<br>205–285 µmol/L	303 (270; 335)<br>239-560<br>54	293 (285; 327)<br>239-385<br>42		
32.	Chlorides,<br>98 – 107 mmol/L	119 (112; 122)<br>61-137	120 (112; 122)<br>62-141		

		54	42		
33.	Lactate<br>0.5-2.22 mmol/L	2.37 (1.91; 2.83)<br>1.28-3.93<br>54	2.47 (2.02; 2.86)<br>1.45-4.23<br>42		
34.	Direct bilirubin<br>0-3.4 µmol/L	3.0 (2.3; 4.1)<br>1.5-13.1<br>54	3.2 (2.5; 4.2)<br>1.5-9.2<br>42		

Comparison of groups was performed by Mann-Whitney test.
*, **, **** - difference from control is statistically significant ($p<0.05$, $p<0.01$, $p<0.0001$)
Abbreviations: ALP - alkaline phosphatase, ALT - alanine aminotransferase, AST - aspartate aminotransferase, CRP – C-reactive protein, GGT - gamma-glutamyltransferase, HDL - high density lipoproteins, LDH - lactate dehydrogenase, SOD – superoxide dismutase, TAS - total antioxidant status.

Table S4. Biochemistry (plate reader). The results of the detailed study of esterase profile are presented as a median and interquartile range. Additionally, the range from minimum to maximum.

	Control (n=37)	OP (n=69)	Post-hoc power	AUC
BChE by ATCh, $\mu mol \times min^{-1} \times L^{-1}$	1628 (1336; 1884)<br>782-2990	1400 (1139; 1760) *<br>716-2653	0.228	0.6228
BChE by BTCh, $\mu mol \times min^{-1} \times L^{-1}$	3136 (2488; 3548)<br>1571-5739	2695 (2334; 3376)<br>1587-5208		
Albumin esterase activity, $\mu mol \times min^{-1} \times L^{-1}$	104.5 (8.6; 167.9)<br>6.0-691.0	134.4 (49.2; 244.6) *<br>6.0-657.8	0.155	0.6173
PON1, $mmol \times min^{-1} \times L^{-1}$	36.3 (28.5; 44.6)<br>15.8-68.6	36.5 (30.4; 40.3)<br>11.7-58.2		

Note: [1] – the minimum activity value is 6 $\mu mol \times min^{-1} \times L^{-1}$
*, **** – the difference from the control group is statistically significant, $p<0.05$, $p<0.0001$
Abbreviations: ATCh – acetylthiocholine, BChE – butyrylcholinesterase, BTCh – butyrylthiocholine, PON1 - paraoxonase-1.

Table S5. Results of chemical analysis with HPLC-MS/MS of biomarkers in the blood of patients. Data are presented as median with interquartile range and range from minimum to maximum.

Biomarker	Control, n=45	OP, n=75	Post-hoc power	AUC
3-Hydroxybutyrate, mM	0.030 (0.021; 0.046)<br>0.005-0.246	0.021 (0.005; 0.045) *<br>0.005-0.483	0.181	0.6182
2-Hydroxybutyrate, mM	0.038 (0.029; 0.056)<br>0.005-0.120	0.023 (0.011; 0.037) ****<br>0.005-0.127	0.503	0.7239
Threonine, mM	0.140 (0.120; 0.170)<br>0.100-0.390	0.140 (0.120; 0.170)<br>0.080-0.290		
Creatine, mM	0.026 (0.014; 0.035)<br>0.001-0.070	0.025 (0.016; 0.040)<br>0.0001-0.099		
3-Methylhistidine, mM	0.026 (0.15; 0.43)<br>0.004-0.081	0.018 (0.008;0.041)<br>0.0001-0.248		
Acetyl-carnitine, mM	0.011 (0.010; 0.013)<br>0.003.-0.028	0.009 (0.004; 0.012) ***<br>0.003-0.021	0.371	0.6856
Uridine, mM	0.003 (0.002; 0.003)<br>0.002-0.007	0.003 (0.002; 0.006)<br>0.002-0.010		
Choline, µM	17.6 (14.0; 22.8)<br>8.2-32.6<br>(n=57)	15.4 (12.8; 20.8)<br>8.2-31.3<br>(n=83)		

*, ***, **** - differences from the control group (Mann-Whitney test) are statistically significant, $p<0.05$, $p<0.001$, $p<0.0001$, respectively.
Note: in samples with 3-hydroxybutyrate and 2-hydroxybutyrate content below the method limit of quantitation (LOQ), the concentration of the analytes was taken equal to the LOQ (0.5 µg/ml or 0.0048 mM), similarly for creatine

(LOQ 10 ng/mL, which corresponds to 0.01 μg/mL or 0.0001 mM) and 3-methylhistidine (LOQ 0.02 μg/mL or 0.0001 mM).

Table S6. Characteristics of FA by the presence, quantity and position of double bonds

	Unsaturated	Saturated
Omega 3	α-Linolenic, C18:3n3<br>Eicosapentaenoic, C20:5n3<br>Docosahexaenoic, C22:6n3	myristic (C14:0),<br>pentadecanoic (C15:0),<br>palmitic (C16:0),<br>margaric (C17:0),<br>stearic (C18:0),<br>arachidic (C20:0),<br>heneicosanoic<br>(C21:0),<br>behenic (C22:0), tricosanoic<br>(C23:0)<br>and lignoceric (C24:0) acids
Omega 6	Linoelaidic, C18:2t<br>Linoleic, C18:2n6c<br>γ-Linolenic, C18:3n6<br>Eicosadienoic, C20:2<br>Eicosatrienoic, C20:3n6<br>Arachidonic, C20:4n6<br>Docosadienoic, C22:2	
Omega 9	Elaidine, C18:1n9t<br>Oleic, C18:1n9c<br>Eicosenoic, C20:1<br>Erucic, C22:1n9<br>Nervonic, C24:1n9	
Others	Myristoleic, C14:1<br>Eicosatrienoic, C20:3n11<br>Pentadecenoic, C15:1<br>Palmitoleic, C16:1n7<br>Heptadecenoic, C17:1	

Table S7. Characteristics of fatty acids depending on chain length and concentration in blood plasma

	Long chain<br>C13-C21	Ultra-long chain<br>C22 and above
Major<br>(more than 60 μg/mL)	Palmitic<br>Stearic<br>Octadecenoic<br>Oleic<br>cis-Octadecadienoic<br>Linoleic	Eicosatetraenoic<br>Arachidonic<br>Docosahexaenoic
Submajor<br>(10-60 μg/mL)	Myristic<br>Margaric<br>trans-Octadecadienoic	Eicosatrienoic acid<br>Eicosapentaenoic acid
Minor<br>(less than 10 μg/mL)	Tridecanoic<br>Myristoleic<br>Pentadecanoic acid<br>Pentadecenoic<br>Palmitoleic<br>Heptadecenoic<br>Elaidic<br>Eicosanoic<br>Arachidic<br>γ-Linolenic<br>Linolelaidic<br>Eicosenoic<br>α-Linolenic<br>Heneicosanoic<br>Eicosadienoic	Behenic<br>Erucic<br>Eicosatrienoic acid<br>Tricosanoic acid<br>Docasadienoic acid<br>Tetracosanoic acid<br>Lignoceric<br>Nervonic

Note: The results for medium-chain minor fatty acids (Octanoic, Decanoic, Undecanoic, Dodecanoic) and Tridecanoic acid were not reliable and were excluded from further consideration.

Table S8. Concentrations (μg/mL) of EFA in the blood plasma of the patients. Results are presented as median, interquartile range, and minimum-to-maximum range.

Name of fatty acid	Control group, n=56	OP group, n=76	Post-hoc power	AUC
Myristic, C14:0	7.43 (5.40; 13.81)<br>1.15-52.01	11.80 (7.20; 19.39) **<br>2.62-116.7	0.254	0.6379
Myristoleic, C14:1	0.62 (0.42; 0.99)<br>0.00-5.72	1.04 (0.62; 1.71) ***<br>0.00-20.43	0.403	0.6763
Pentadecanoic, C15:0	3.63 (2.92; 5.35)<br>0.80-8.32	3.50 (2.54; 5.35)<br>0.79-12.86		
Pentadecenoic, C15:1	0.00 (0.00; 0.12)<br>0.00-1.32	0.00 (0.00; 0.17)<br>0.00-0.56		
Palmitic, C16:0	311 (241; 438)<br>147-1222	301 (218; 378)<br>67-1275		
Palmitoleic, C16:1n-7	22.8 (13.8; 32.9)<br>3.25-170.30	26.3 (18.4; 44.2)<br>2.80-182.30		
Margaric, C17:0	8.25 (5.87; 9.70)<br>1.00-19.91	5.51 (4.30; 8.44) **<br>1.07-30.15	0.293	0.6490
Heptadecenoic, C17:1	2.05 (1.45; 3.03)<br>0.84-12.53	2.44 (1.54; 4.00)<br>0.00-12.32		
Stearic, C18:0	178 (137; 251)<br>67-594	138 (105; 185) **<br>30-827	0.313	0.6544
Elaidic, C18:1n9t	4.34 (2.42; 7.04)<br>0.40-62.90	3.89 (2.32; 6.99)<br>0.75-128.70		
Oleic, C18:1n9c	199 (144; 309)<br>75-1260	254 (156; 342)<br>0.3-1264		
Linoelaidic, C18:2t	1.60 (1.09; 2.21)<br>0.00-3.96	1.59 (1.19; 2.26)<br>0.00-5.15		
Linoleic, C18:2n6c	331 (256; 419)<br>135-1462	310 (241; 375)<br>1-1477		
Arachidic, C20:0	1.36 (1.19; 1.83)<br>0.77-3.77	1.34 (1.10; 1.90)<br>0.68-8.47		
γ-Linolenic, C18:3n6	8.53 (4.79; 12.40)<br>0.00-42.34	8.78 (4.78; 14.17)<br>1.08-34.52		
Eicosenoic, C20:1	2.91 (0.53; 5.01)<br>0.00-7.86	2.41 (0.92; 3.77)<br>0.00-10.33		
α-Linolenic, C18:3n3	6.65 (4.90; 11.07)<br>0.00-61.86	7.52 (5.06; 11.75)<br>0.00-57.81		
Heneicosanoic, C21:0	0.00 (0.00; 0.00)<br>0.00-0.00	0.00 (0.00; 0.00)<br>0.00-0.75		
Eicosadienoic, C20:2	10.78 (8.40; 14.86)<br>1.17-34.02	8.60 (5.96; 11.98) **<br>1.23-49.23	0.258	0.6332
Behenic, C22:0	0.90 (0.00; 1.02)<br>0.00-2.50	0.95 (0.86; 1.08)<br>0.00-1.88		
cis-8,11,14-Eicosatrienoic, C20:3n6	85.0 (64.3; 119.5)<br>12.7-237.3	56.5 (38.8; 96.0) **<br>3.8-516.8	0.333	0.6579
Erucic, C22:1n9	0.78 (0.00; 0.91)<br>0.00-1.72	0.87 (0.73; 1.31) **<br>0.00-5.76	0.335	0.6659
cis-11,14,17-Eicosatrienoic, C20:3n11	1.28 (1.13; 1.78)<br>1.00-102.00	1.26 (1.10; 1.58)<br>0.97-56.52		
Arachidonic, C20:4n6	387 (298; 549)	283 (196; 407) ***	0.385	0.6732

Name of fatty acid	Control group, n=56	OP group, n=76	Post-hoc power	AUC
	107-1258	61-1983		
Tricosanoic, C23:0	0.00 (0.00; 0.00)<br>0.00-1.19	0.00 (0.00; 0.00)<br>0.00-1.02		
Docosadienoic, C22:2	0.00 (0.00; 1.00)<br>0.00-17.36	0.00 (0.00; 1.05)<br>0.00-8.02		
Lignoceric, C24:0	0.00 (0.00; 0.00)<br>0.00-1.73	0.00 (0.00; 0.00)<br>0.00-2.11		
Eicosapentaenoic, C20:5	52.0 (26.0; 79.4)<br>1.6-186.2	27.2 (16.8; 74.0) *<br>5.2-369.1	0.167	0.6041
Nervonic, C24:1n9	1.01 (0.00; 1.19)<br>0.00-2.24	1.00 (0.00; 1.15)<br>0.00-2.93		
Docosahexaenoic, C22:6n3	608 (342; 773)<br>27-1817	248 (161; 534) ****<br>17-2460	0.650	0.7444

*, **, ***, **** - differences from the control group (Mann-Whitney test) are statistically significant, p<0.05, p<0.01, p<0.001, p<0.0001, respectively.

Table S9. Concentrations (μg/mL) of NEFA in the blood plasma of the subjects. Results are presented as median, interquartile range, and minimum-to-maximum range.

Name of fatty acid	Control group, n=56	OP group, n=76	Post-hoc power	AUC
Myristic, C14:0	7.09 (5.94; 8.73)<br>3.52-15.72	9.77 (7.03; 14.35) ****<br>3.29-68.75	0.512	0.7072
Myristoleic, C14:1	1.36 (0.92; 1.93)<br>0.00-13.28	1.38 (0.94; 2.01)<br>0.00-21.21		
Pentadecanoic, C15:0	2.58 (2.15; 3.03)<br>0.00-4.56	2.85 (2.37; 3.50) *<br>0.00-6.67	0.175	0.6063
Pentadecenoic, C15:1	0.00 (0.00; 0.00)<br>0.00-0.76	0.00 (0.00; 0.00)<br>0.00-1.24		
Palmitic, C16:0	139 (99; 171)<br>72-314	172 (134; 229) ***<br>51-507	0.381	0.6827
Palmitoleic, C16:1n-7	12.09 (8.16; 17.76)<br>2.92-34.06	14.51 (10.15; 24.19) *<br>1.59-118.60	0.184	0.6095
Margaric, C17:0	3.05 (2.48; 3.57)<br>0.75-6.42	3.34 (2.86; 4.33) **<br>1.25-3.19	0.267	0.6392
Heptadecenoic, C17:1	0.84 (0.00; 1.41)<br>0.00-38.12	1.44 (0.95; 5.22) ****<br>0.00-85.00	0.543	0.7177
Stearic, C18:0	63.1 (49.1; 79.8)<br>31.2-127.6	75.9 (61.0; 93.2) **<br>23.9-177.0	0.267	0.6403
Elaidic, C18:1n9t	1.44 (1.01; 2.74)<br>0.47-4.52	1.91 (1.26; 2.95)<br>0.44-192.4		
Oleic, C18:1n9c	119.8 (86.56; 153.2)<br>55.5-275.6	152.3 (108.1; 199.2) **<br>0.45-534.1	0.323	0.6555
Linoelaidic, C18:2t	1.08 (0.93; 1.34)<br>0.00-1.86	1.32 (1.06; 1.80) **<br>0.00-1.86	0.108	0.6480
Linoleic, C18:2n6c	140 (100; 172)<br>34-333	175 (126; 226) **<br>2-426	0.313	0.6525
Arachidic, C20:0	1.13 (1.01; 1.30)<br>0.57-1.82	1.11 (1.00; 1.31)<br>0.00-3.54		
γ-Linolenic, C18:3n6	3.15 (2.17; 4.35)<br>0.00-16.19	4.68 (2.64; 7.27) **<br>0.00-19.42	0.319	0.6548
Eicosenoic, C20:1	1.20 (0.00; 1.80)<br>0.00-3.00	1.64 (0.85; 2.13) **<br>0.00-4.77	0.311	0.6518

α-Linolenic, C18:3n3	4.21 (3.15; 5.39)<br>1.40-8.80	4.74 (3.49; 6.62)<br>1.20-32.97		
Heneicosanoic, C21:0	0.00 (0.00; 0.65)<br>0.00-0.80	0.00 (0.00; 0.67)<br>0.00-1.20		
Eicosadienoic, C20:2	3.15 (2.44; 4.09)<br>1.60-6.86	4.04 (3.16; 5.16) ***<br>1.03-12.48	0.388	0.6741
Behenic, C22:0	1.00 (0.93; 1.09)<br>0.00-1.86	1.02 (0.95; 1.10)<br>0.00-2.13		
cis-8,11,14-Eicosatrienoic, C20:3n6	14.4 (10.6; 21.7)<br>2.0-56.8	22.4 (16.3; 33.0) ***<br>2.5-69.3	0.420	0.6828
Erucic, C22:1n9	0.77 (0.15; 1.07)<br>0.00-3.80	0.88 (0.71; 1.09)<br>0.00-7.20		
cis-11,14,17-Eicosatrienoic, C20:3n11	1.05 (0.97; 1.19)<br>0.00-22.66	1.10 (0.99; 1.93)<br>0.00-105.20		
Arachidonic, C20:4n6	90.4 (58.8; 132.0)<br>33.6-273.2	116.0 (88.8; 178.6) **<br>20.6-297.0	0.325	0.6567
Tricosanoic, C23:0	0.00 (0.00; 0.00)<br>0.00-0.88	0.00 (0.00; 0.00)<br>0.00-1.95		
Docosadienoic, C22:2	0.00 (0.00; 0.90)<br>0.00-1.44	0.00 (0.00; 0.99)<br>0.00-2.24		
Lignoceric, C24:0	0.00 (0.00; 1.35)<br>0.00-2.88	0.00 (0.00; 0.00)<br>0.00-3.26		
Eicosapentaenoic, C20:5	10.21 (6.66; 15.63)<br>2.09-48.84	15.90 (7.99; 23.56) *<br>2.39-132.30	0.199	0.6157
Nervonic, C24:1n9	0.97 (0.00; 1.05)<br>0.00-2.00	0.00 (0.00; 1.05)<br>0.00-2.36		
Docosahexaenoic, C22:6n3	95 (65; 127)<br>24-322	142 (81; 239) **<br>15-533	0.325	0.6558

*, **, ***, **** - differences from the control group (Mann-Whitney test) are statistically significant, p<0.05, p<0.01, p<0.001, p<0.0001, respectively.

Table S10. Concentrations (μg/mL) of the main classes of esterified and non-esterified fatty acids in the blood plasma of the patients. Results are presented as median, interquartile range, and minimum-to-maximum range.

Name of fatty acid	Control group, n=56	OP group, n=76	Post-hoc power	AUC
Esterified major FA	1979 (1502; 2811)<br>774-5788	1523 (1233; 2100) **<br>176-9287	0.313	0.6527
Esterified submajor FA	172.0 (114.7; 205.0)<br>39.1-399.2	114.5 (70.5; 213.5) *<br>24.4-934.3	0.236	0.6288
Esterified minor FA	71.1 (52.9; 101.2)<br>32.4-435.2	82.5 (53.9; 110.8)<br>19.7-336.3		
Esterified long chain FA (C13-C21)[1]	1126 (851; 1556)<br>505-5003	1118 (788; 1372)<br>121-5121		
Esterified ultra-long chain fatty acids (C22 and longer)	1187 (777; 1516)<br>169-3272	613 (442; 1100) ****<br>101-5344	0.574	0.7232
Esterified saturated [3]	515 (396; 721)<br>239-1897	472 (346; 600)<br>107-2169		
Esterified unsaturated	1714 (1338; 2342)<br>618-4725	1230 (991; 1799) **<br>115-8297	0.352	0.6638
Esterified n3/n6	0.72 (0.53; 0.99)<br>0.10-1.35	0.51 (0.31; 0.76) ****<br>0.09-1.28	0.496	0.7028
Esterified n3/(n6+n9)	0.58 (0.42; 0.78)<br>0.07-1.14	0.39 (0.21; 0.59) ****<br>0.06-1.00	0.554	0.7183

Esterified n3/(all FA)	0.27 (0.22; 0.34)<br>0.04-0.44	0.20 (0.12; 0.28) ****<br>0.04-0.41	0.574	0.7223
Non-esterified major FA	633 (472;824)<br>324-1610	816 (635; 1138) ***<br>169-2075	0.383	0.6725
Non-esterified submajor FA	36.0 (27.8; 50.9)<br>15.1-93.2	52.0 (37.7; 76.4) ***<br>11.4-204.9	0.451	0.6906
Non-esterified minor FA	37.3 (31.6; 47.7)<br>21.6-83.2	59.0 (37.0; 105.9) ****<br>14.8-259.5	0.554	0.7179
Non-esterified long chain FA (C13-C21)[2]	507 (367; 619)<br>264-1146	622 (490; 836) ***<br>148-1753	0.394	0.6758
Non-esterified ultra-long chain FA (C22 and longer)	205 (168; 295)<br>84-632	322 (219; 492) ***<br>47-872	0.431	0.6859
Non-esterified saturated FA[4]	227 (163; 265)<br>116-468	266 (215; 350) ***<br>82-764	0.398	0.6776
Non-esterified unsaturated	485 (371; 643)<br>248-1309	700 (505; 1018) ***<br>113-1827	0.465	0.6954
Non-esterified n3/n6	0.46 (0.24; 0.58)<br>0.18-0.90	0.49 (0.38; 0.60)<br>0.13-2.06		
Non-esterified n3/(n6+n9)	0.31 (0.22; 0.39)<br>0.11-0.66	0.33 (0.25; 0.40)<br>0.08-0.89		
Non-esterified n3/(all FA)	0.16 (0.12; 0.19)<br>0.07-0.29	0.17 (0.13; 0.20)<br>0.05-0.33		

Notes: [1,2] The sum of long-chain fatty acids was calculated without tridecanoic acid, since the results of its measurement are not reliable.

[3.4] The sum of SFA included the results of determination of 10 analytes, namely myristic (C14:0), pentadecanoic (C15:0), palmitic (C16:0), margaric (C17:0), stearic (C18:0), arachidic (C20:0), heneicosanoic (C21:0), behenic (C22:0), tricosanoic (C23:0) and lignoceric (C24:0) acids;

*, **, ***, **** - differences from the control group (Mann-Whitney test) are statistically significant, p<0.5, p<0.01, p<0.001, p<0.0001, respectively.

Table S11. Correlation pairs of the most reliable statistically significant indicators.

Indicator 1	Indicator 2	Spearman's correlation coefficient with confidence interval and statistical significance of this relationship		
		The entire array (Control + OP)	Control	OP
NE Myristic	NE Heptadecenoic	0.62<br>0.49 to 0.71<br>p <0.0001	0.39<br>0.14 to 0.60<br>p=0.0029	0.61<br>0.44 to 0.74<br>p <0.0001
NE Heptadecenoic	TAS	0.52<br>0.36 to 0.65<br>p <0.0001	0.34<br>0.030 to 0.59<br>p=0.028	0.53<br>0.33 to 0.68<br>p <0.0001
E Docosahexaenoic	LDH	0.64<br>0.53 to 0.74<br>p <0.0001	0.22<br>-0.06 to 0.46<br>p=0.107	0.67<br>0.51 to 0.78<br>p <0.0001
E Docosahexaenoic	TAS	-0.55<br>-0.67 to -0.403<br><0.0001	-0.23<br>-0.51 to 0.09<br>p=0.144	-0.47<br>-0.64 to -0.26<br><0.0001
LDH	TAS	-0.56<br>-0.67 to -0.42<br>p <0.0001	-0.02<br>-0.33 to 0.28<br>p=0.878	-0.61<br>-0.73 to -0.44<br>p <0.0001

Abbreviations: E – esterified, NE - non-esterified, TAS - total antioxidant status, LDH - lactate dehydrogenase

Table S12. Correlation pairs of esterified docosahexaenoic fatty acid, LDH activity and TAS concentration with other statistically significant parameters.

Indicator 1	Indicator 2	Spearman's correlation coefficient with confidence interval and statistical significance of this relationship		
		The entire array (Control + OP)	Control	OP
E Docosahexaenoic	Ca	-0.65<br>-0.74 to -0.53<br>p <0.0001	-0.40<br>-0.60 to -0.14<br>p=0.0024	-0.66<br>-0.77 to -0.50<br>p <0.0001
E Docosahexaenoic	E Margaric	0.68<br>0.57 to 0.76<br>p <0.0001	0.53<br>0.30 to 0.70<br>p <0.0001	0.70<br>0.55 to 0.80<br>p <0.0001
E Docosahexaenoic	E Eicosadienoic	0.70<br>0.60 to 0.78<br>p <0.0001	0.60<br>0.40 to 0.75<br>p <0.0001	0.70<br>0.56 to 0.80<br>p <0.0001
E Docosahexaenoic	E cis-8,11,14-Eicosatrienoic	0.77<br>0.69 to 0.84<br>p <0.0001	0.58<br>0.37 to 0.74<br>p <0.0001	0.77<br>0.65 to 0.85<br>p <0.0001
E Docosahexaenoic	E Arachidonic	0.85<br>0.79 to 0.89<br>p <0.0001	0.75<br>0.60 to 0.85<br>p <0.0001	0.83<br>0.74 to 0.90<br>p <0.0001
E Docosahexaenoic	E Eicosapentaenoic	0.76<br>0.67 to 0.827<br>p <0.0001	0.60<br>0.39 to 0.75<br>p <0.0001	0.78<br>0.66 to 0.85<br>p <0.0001
LDH	Ca	-0.53<br>-0.65 to -0.40<br>p <0.0001	-0.05<br>-0.31 to 0.22<br>p=0.7187	-0.67<br>-0.78 to -0.53<br>p <0.0001
LDH	ALP	0.49<br>0.35 to 0.61<br>p <0.0001	0.14<br>-0.13 to 0.39<br>p=0.3049	0.64<br>0.49 to 0.76<br>p <0.0001
TAS	Ca	0.63<br>0.49 to 0.72<br>p <0.0001	0.32<br>0.02 to 0.57<br>p=0.0323	0.63<br>0.47 to 0.75<br>p <0.0001

Abbreviations: E – esterified, ALP - alkaline phosphatase, LDH - lactate dehydrogenase, TAS - total antioxidant status.

Table S13. Correlation pairs of other statistically significant indicators.

Indicator 1	Indicator 2	Spearman's correlation coefficient with confidence interval and statistical significance of this relationship		
		The entire array (Control + OP)	Control	OP
E Myristoleic	E Myristic	0.91<br>0.88 to 0.94	0.85<br>0.76 to 0.91	0.94<br>0.90 to 0.96
E Arachidonic	E Docosahexaenic	0.85<br>0.79 to 0.89	0.75<br>0.60 to 0.85	0.83<br>0.74 to 0.89
E Arachidonic	E cis-8,11,14-Eicosatrienoic	0.81<br>0.74 to 0.86	0.70<br>0.52 to 0.81	0.80<br>0.690 to 0.87
NE Palmitic	NE Myristic	0.78<br>0.70 to 0.84	0.63<br>0.43 to 0.77	0.82<br>0.72 to 0.88
NE Margaric	NE Myristic	0.79<br>0.71 to 0.85	0.75<br>0.61 to 0.85	0.81<br>0.71 to 0.88
NE Margaric	NE Palmitic	0.82<br>0.76 to 0.87	0.78<br>0.64 to 0.87	0.83<br>0.73 to 0.89
NE Stearic	NE Palmitic	0.93	0.93	0.93

		0.90 to 0.95	0.89 to 0.96	0.89 to 0.95
NE Stearic	NE Margaric	0.82<br>0.75 to 0.87	0.74<br>0.58 to 0.84	0.85<br>0.77 to 0.91
NE Oleic	NE Palmitic	0.93<br>0.91 to 0.95	0.90<br>0.83 to 0.94	0.95<br>0.93 to 0.97
NE Oleic	NE Margaric	0.79<br>0.71 to 0.85	0.72<br>0.56 to 0.83	0.81<br>0.70 to 0.87
NE Oleic	NE Stearic	0.85<br>0.79 to 0.89	0.79<br>0.66 to 0.87	0.88<br>0.82 to 0.93
NE Linoleic	NE Palmitic	0.89<br>0.84 to 0.92	0.95<br>0.91 to 0.97	0.84<br>0.76 to 0.90
NE Linoleic	NE Stearic	0.83<br>0.77 to 0.88	0.90<br>0.84 to 0.94	0.78<br>0.67 to 0.86
NE Linoleic	NE Oleic	0.84<br>0.78 to 0.88	0.86<br>0.78 to 0.92	0.83<br>0.73 to 0.89
NE Eicosenoic	NE Eicosadienoic	0.69<br>0.59 to 0.78	0.45<br>0.20 to 0.64	0.82<br>0.73 to 0.88
NE Eicosadienoic	NE Palmitic	0.89<br>0.84 to 0.92	0.92<br>0.87 to 0.96	0.84<br>0.75 to 0.90
NE Eicosadienoic	NE Margaric	0.83<br>0.76 to 0.88	0.80<br>0.68 to 0.88	0.82<br>0.73 to 0.88
NE Eicosadienoic	NE Stearic	0.85<br>0.80 to 0.90	0.88<br>0.80 to 0.93	0.82<br>0.73 to 0.88
NE Eicosadienoic	NE Oleic	0.86<br>0.80 to 0.90	0.85<br>0.76 to 0.91	0.84<br>0.76 to 0.90
NE Eicosadienoic	NE Linoleic	0.86<br>0.80 to 0.90	0.91<br>0.85 to 0.95	0.81<br>0.71 to 0.87
NE Eicosatrienoic	NE Palmitic	0.82<br>0.75 to 0.87	0.79<br>0.66 to 0.87	0.81<br>0.72 to 0.88
NE Eicosatrienoic	NE Stearic	0.80<br>0.73 to 0.86	0.79<br>0.66 to 0.87	0.79<br>0.69 to 0.86
NE Eicosatrienoic	NE Eicosadienoic	0.83<br>0.76 to 0.88	0.80<br>0.67 to 0.88	0.82<br>0.72 to 0.88
NE Arachidonic	NE Palmitic	0.84<br>0.78 to 0.88	0.75<br>0.60 to 0.85	0.84<br>0.75 to 0.89
NE Arachidonic	NE Stearic	0.83<br>0.77 to 0.88	0.80<br>0.68 to 0.88	0.81<br>0.71 to 0.88
NE Docosahexaenoic	NE Arachidonic	0.79<br>0.71 to 0.85	0.62<br>0.42 to 0.76	0.82<br>0.73 to 0.89

Note: All correlation coefficients listed in the table are statistically significant, $p<0.0001$
